# Infection of neonatal mice with the murine norovirus strain WU23 is a robust model to study norovirus pathogenesis

**DOI:** 10.1038/s41684-023-01166-5

**Published:** 2023-05-04

**Authors:** Amy M. Peiper, Emily W. Helm, Quyen Nguyen, Matthew Phillips, Caroline G. Williams, Dhairya Shah, Sarah Tatum, Neha Iyer, Marco Grodzki, Laura B. Eurell, Aqsa Nasir, Megan T. Baldridge, Stephanie M. Karst

**Affiliations:** 1 Department of Molecular Genetics & Microbiology, College of Medicine, University of Florida, Gainesville, FL USA; 2 Office of Research, College of Medicine, University of Florida, Gainesville, FL USA; 3 Department of Pathology, Immunology and Laboratory Medicine, College of Medicine, University of Florida, Gainesville, FL USA; 4 Division of Infectious Diseases, Department of Medicine, Edison Family Center for Genome Sciences and Systems Biology, Washington University School of Medicine, St. Louis, MO USA

**Keywords:** Virology, Infectious diseases

## Abstract

Noroviruses are the leading cause of severe childhood diarrhea and foodborne disease worldwide. While they are a major cause of disease in all age groups, infections in the very young can be quite severe, with annual estimates of 50,000–200,000 fatalities in children under 5 years old. In spite of the remarkable disease burden associated with norovirus infections, very little is known about the pathogenic mechanisms underlying norovirus diarrhea, principally because of the lack of tractable small animal models. The development of the murine norovirus (MNV) model nearly two decades ago has facilitated progress in understanding host–norovirus interactions and norovirus strain variability. However, MNV strains tested thus far either do not cause intestinal disease or were isolated from extraintestinal tissue, raising concerns about translatability of research findings to human norovirus disease. Consequently, the field lacks a strong model of norovirus gastroenteritis. Here we provide a comprehensive characterization of a new small animal model system for the norovirus field that overcomes prior weaknesses. Specifically, we demonstrate that the WU23 MNV strain isolated from a mouse naturally presenting with diarrhea causes a transient reduction in weight gain and acute self-resolving diarrhea in neonatal mice of several inbred mouse lines. Moreover, our findings reveal that norovirus-induced diarrhea is associated with infection of subepithelial cells in the small intestine and systemic spread. Finally, type I interferons (IFNs) are critical to protect hosts from norovirus-induced intestinal disease whereas type III IFNs exacerbate diarrhea. This latter finding is consistent with other emerging data implicating type III IFNs in the exacerbation of some viral diseases. This new model system should enable a detailed investigation of norovirus disease mechanisms.

## Abstract (web summary)

A mouse model to study norovirus pathogenesis is described. The murine norovirus strain WU23, originally isolated from an intestinal site, induces severe but self-resolving diarrhea in neonates, reflecting the pathology observed during human norovirus infection.

## Main

Human noroviruses are the leading cause of severe childhood diarrhea and gastroenteritis outbreaks globally, responsible for an estimated 685 million total cases and 50,000–200,000 deaths in young children each year^1–4^. Understanding the pathogenesis of noroviruses, particularly in young hosts, is thus an important priority to aid in the development of vaccines and therapeutics. Yet very little is known about the mechanisms underlying human norovirus disease due to a lack of robust symptomatic in vivo model systems. While certain large animals develop mild diarrhea following human norovirus infection^5–7^, a genetically tractable small animal model of self-resolving acute gastroenteritis is critical to advance our understanding of norovirus pathogenesis. The first murine norovirus (MNV1) was isolated from the brain of an immunodeficient mouse in 2003 and was shown to be infectious orally and shed in the feces^8^. Since then, numerous other genetically similar but phenotypically distinct murine norovirus (MNV) strains have been discovered (for example, refs. ^9,10^). Although all MNV strains characterized so far use the common host receptor CD300lf (refs. ^11–13^), there are striking differences in their in vivo pathogenesis. For example, MNV1 infects intestinal immune cells while CR6 infects tuft cells despite sharing 87% genetic identity and using the same host receptor^14–16^. This virus strain variability in cellular tropism may extend to human noroviruses since both epithelial and immune cell infection have been reported in various animal model and human biopsy studies^5,17–21^. Yet the pathophysiological mechanisms of norovirus-induced diarrhea remain undefined.

In an effort to develop a small animal model of symptomatic norovirus infection to probe key aspects of norovirus pathogenesis, we recently demonstrated that MNV1 causes acute self-resolving diarrhea in neonatal wild-type mice^22^. This is consistent with increased disease severity of human norovirus infection in infants and children compared with adults^4,23–25^. While MNV1 intestinal disease in neonatal mice closely mirrors human disease, questions remain about its relevance in modeling human norovirus infection due to its original isolation from the brain. It has been postulated that MNV1 may contain adaptive mutations facilitating its systemic spread from the intestinal tract and that this dissemination is not a common feature of norovirus infections. This is supported by the finding that other commonly studied MNV strains isolated from intestinal sites (for example, CR6 and MNV3) do not disseminate extraintestinally as readily as MNV1 (refs. ^26,27^). Paradoxically though, CR6 and MNV3 are less virulent in neonatal wild-type mice and adult interferon (IFN)-deficient mice compared with MNV1 (refs. ^22,26,27^). In this Article, we report that a MNV strain called WU23 originally isolated from an intestinal site^10^ is diarrheagenic and disseminates extraintestinally, thus sharing key properties with MNV1. Both diarrheagenic MNV1 and WU23 target subepithelial cells in intestinal tissue and cells in extraintestinal tissues, but not intestinal epithelial cells. These findings establish a positive relationship between subepithelial cell infection and norovirus induction of the hallmark symptom of diarrhea. Additional support for the importance of subepithelial cell infection in norovirus disease is provided by the nature of the host response: type I IFNs protect subepithelial cells from intestinal virus infections while type III IFNs protect mucosal surfaces. In neonatal mice infected with diarrheagenic WU23, type I IFNs were critical to prevent severe disease while type III IFNs exacerbated disease. Collectively, this small animal model represents a strong platform for understanding how noroviruses cause diarrhea.

## Results

### WU23 shares a tissue tropism with virulent MNV1 for the small intestine and extraintestinal tissues

**Fig. 1:**
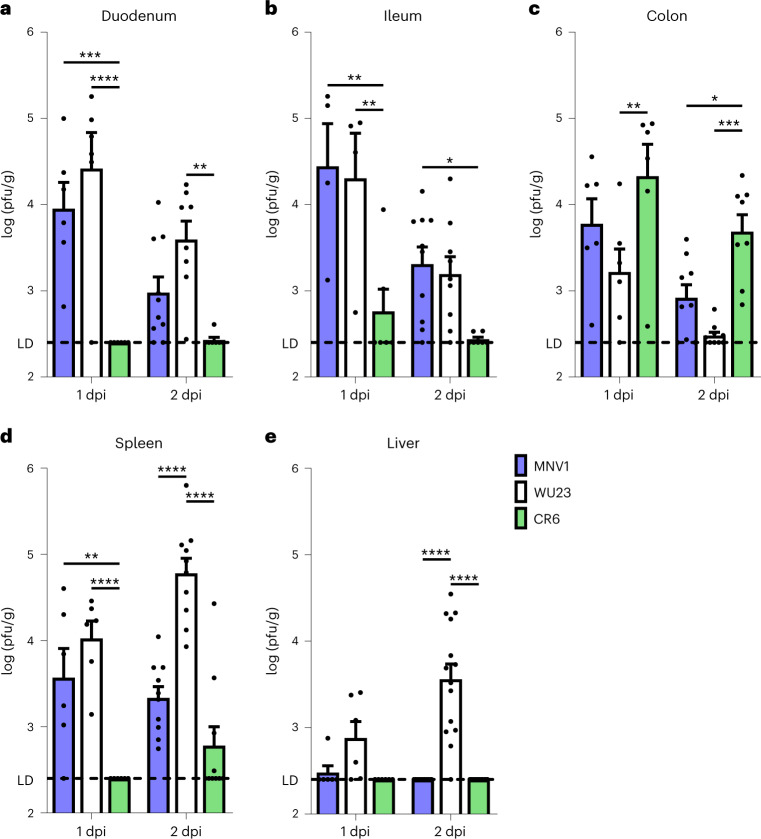
WU23 tissue tropism mirrors acute MNV1 tropism in adult mice. **a**–**e**, Groups of adult C57BL/6J (B6) mice were p.o. infected with 10^7^ TCID_50_ units of WU23, MNV1 or CR6. At 1 (*n* = 6 per condition) or 2 (*n* = 7–10 per condition) dpi, viral titers were determined in the duodenum (**a**), ileum (**b**), colon (**c**), spleen (**d**) and liver (**e**) by plaque assay (pfu, plaque forming unit). Data are presented as mean ± s.e.m.; *P* values were determined using two-sided ANOVA with corrections for multiple comparisons using Tukey’s test. *P < 0.05, **P < 0.01, ***P < 0.001 and ****P < 0.0001. Dashed line indicates limit of detection (LD).

Multiple MNV strains are commonly studied, including acute, virulent MNV1 and persistent, avirulent CR6 and MNV3 strains^8–10,22,26,27^. Yet none of these viruses was isolated from a host naturally presenting with the hallmark norovirus symptoms of gastroenteritis. In an effort to refine this model system to reflect human norovirus pathogenesis as closely as possible, we were compelled to test the virulence of a MNV strain isolated from a mouse naturally presenting with diarrhea. Several such strains were reported in 2007 (ref. ^10^), but they have not been extensively characterized. One of these strains, referred to as WU23, was recently demonstrated to use the same receptor as other commonly studied MNV strains, CD300lf (ref. ^11^); we therefore selected this strain for further characterization. In spite of using a common receptor, it is well established that MNV strains display regionally variable patterns of intestinal infection in adult mice: MNV1 preferentially infects the small intestine, while MNV3 and CR6 preferentially infect the colon^28–30^. Furthermore, MNV1 disseminates to the spleen more readily than other well-studied MNV strains^26,27^, an observation that has been suggested to be linked to its original isolation from a systemic site^8^. Thus, it was of interest to compare the tissue tropism of WU23 with prototype strains MNV1 and CR6 in adult mice. Intestinal titers of WU23 mirrored MNV1, with high titers in the small intestine (Fig. 1a,b) and lower titers in the colon (Fig. 1c). Strikingly, peak WU23 splenic titers were 1.5 and 2 logs higher than MNV1 or CR6 titers, respectively (Fig. 1d), revealing that dissemination to extraintestinal tissues is not unique to MNV1 but is also a property of a virus isolated from an enteric site. This is consistent with a prior study by Graziano et al. that detected WU23 genomic RNA in the spleen at 7 days post-infection (dpi)^11^. Further underscoring this finding, WU23 also reached appreciable titers in the liver at 2 dpi (Fig. 1e). Overall, a MNV strain isolated from the feces of a mouse naturally presenting with diarrhea infects the small intestine and extraintestinal tissues, dispelling the notion that MNV1 dissemination represents a nonphysiological feature of norovirus infection. However, like other MNV strains, WU23 did not cause overt disease in wild-type adult mice (Supplementary Fig. 1).

### C57BL/6 (B6) and BALB/c neonatal mice are comparably susceptible to WU23-induced diarrhea

**Fig. 2:**
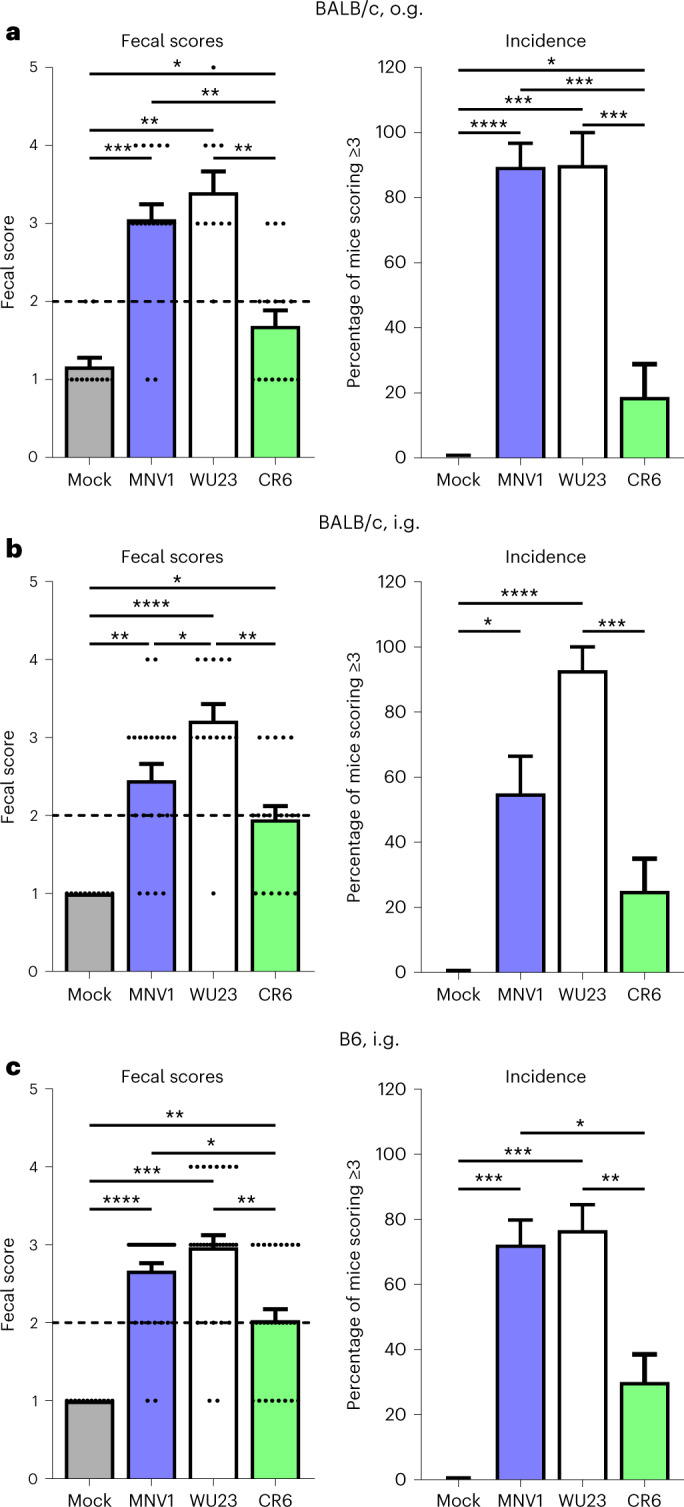
Neonatal BALB/c and B6 mice are comparably susceptible to MNV diarrhea. **a**, Groups of P3 BALB/c pups were infected by o.g. with 10^7^ TCID_50_ units of MNV1 (*n* = 19), WU23 (*n* = 10), CR6 (*n* = 16) or mock inoculum (*n* = 12). At 2 dpi, fecal consistency was determined by palpating their abdomens. The proportion of mice scoring a 3 or above is presented as incidence of diarrhea. **b**, Groups of P3 BALB/c pups were infected by i.g. with 10^7^ TCID_50_ units of MNV1 (*n* = 20), WU23 (*n* = 14), CR6 (*n* = 20) or mock inoculum (*n* = 11). At 2 dpi, fecal consistency was determined by palpating their abdomens. The proportion of mice scoring a 3 or above is presented as incidence of diarrhea. **c**, Groups of P3 C57BL/6J (B6) pups were infected by i.g. with 10^7^ TCID_50_ units of MNV1 (*n* = 36), WU23 (*n* = 30), CR6 (*n* = 30) or mock inoculum (*n* = 12). At 2 dpi, fecal consistency was determined by palpating their abdomens. Mice from at least three independent litters per condition were analyzed in all panels. Mice that did not defecate were excluded from analysis. The proportion of mice scoring a 3 or above is presented as incidence of diarrhea. Data are presented as mean ± s.e.m.; *P* values were determined using two-sided ANOVA with corrections for multiple comparisons using Tukey’s test. *P < 0.05, **P < 0.01, ***P < 0.001 and ****P < 0.0001. Dashed line highlights scores reflective of baseline fecal consistency (≤2) and diarrhea (>2).

**Fig. 3:**
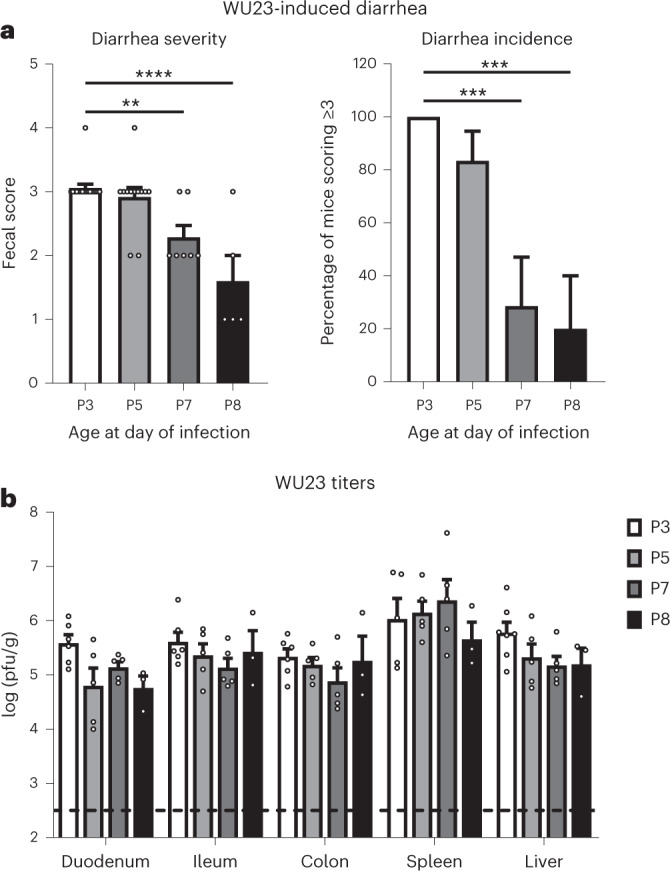
WU23 diarrhea is tightly age-restricted in B6 mice. Groups of C57BL/6J (B6) pups at P3 (*n* = 17), P5 (*n* = 12), P7 (*n* = 7) or P8 (*n* = 5) were infected by i.g. with 10^7^ TCID_50_ units of WU23. **a**, At 2 dpi, fecal consistency was determined by palpating their abdomens. Mice that did not defecate were excluded from analysis. The proportion of mice scoring a 3 or above is presented as incidence of diarrhea. Mice from at least two independent litters per condition were analyzed. **b**, At 2 dpi, viral titers were determined in the indicated segments of the intestinal tract, spleen and liver by plaque assay. At least three mice per condition were analyzed. Data are presented as mean ± s.e.m.; *P* values were determined using two-sided ANOVA with corrections for multiple comparisons using Tukey’s test. *P < 0.05, **P < 0.01, ***P < 0.001 and ****P < 0.0001. Dashed line indicates limit of detection.

On the basis of our prior study demonstrating that MNV1 induces diarrhea in neonatal BALB/c mice^22^, we next sought to examine the virulence of WU23 in this model. Three-day-old (P3) BALB/c pups infected with 10^7^ 50% tissue culture infectious dose (TCID_50_) units WU23 by oral gavage (o.g.) developed diarrhea of comparable severity and incidence to MNV1 (Fig. 2a). As previously reported^22^, CR6 caused significantly reduced diarrhea compared with MNV1 under these conditions. Because most knockout mouse strains are on the B6 background, it was of interest to determine whether B6 neonates are also susceptible to MNV-induced diarrhea. We attempted to infect P3 and P4 B6 mice by o.g. as we had done in BALB/c pups, but this inoculation method was traumatic in the B6 neonates, probably due to their relatively smaller size at this age. This was not related to MNV infection since comparable trauma was observed in mock- and MNV1-inoculated pups. To overcome this limitation, we tested whether neonatal mice were susceptible to MNV-induced diarrhea using intragastric inoculation (i.g.) since this inoculation procedure can be performed on smaller pups. P3 BALB/c mice infected by i.g. with MNV1 or WU23 developed diarrhea that was of comparable severity and incidence to pups infected by o.g. (Fig. 2b). CR6 also induced statistically significant fecal inconsistency under these conditions, although it was less severe than disease caused by other viruses, as reflected by a reduced proportion of mice scoring 3 or higher, which is considered diarrhea when calculating incidence of disease (Fig. 2b). Using this method, all three MNV strains induced diarrhea in P3 B6 mice, with a significantly increased incidence of disease in MNV1- and WU23-infected pups compared with CR6-infected pups (Fig. 2c). We previously observed that MNV1-induced diarrhea in BALB/c pups was tightly age restricted. To determine whether the same restriction occurs in B6 neonates, B6 pups were infected with 10^7^ TCID_50_ units WU23 at P3, P5, P7 or P8 and diarrhea assessed at 2 dpi. While P5 pups developed diarrhea comparable to P3 pups, P7 and P8 pups developed less severe diarrhea (Fig. 3a). Interestingly, this age restriction was not due to a reduced susceptibility to infection since virus titers were comparable in all groups (Fig. 3b). Overall, these findings demonstrate that neonatal BALB/c and B6 mice are comparably susceptible to MNV-induced diarrhea, expanding the model system to enable investigation in commonly available knockout mouse lines. Moreover, they reveal that WU23 is diarrheagenic.

### Virulence correlates with sustained intestinal and extraintestinal replication

**Fig. 4:**
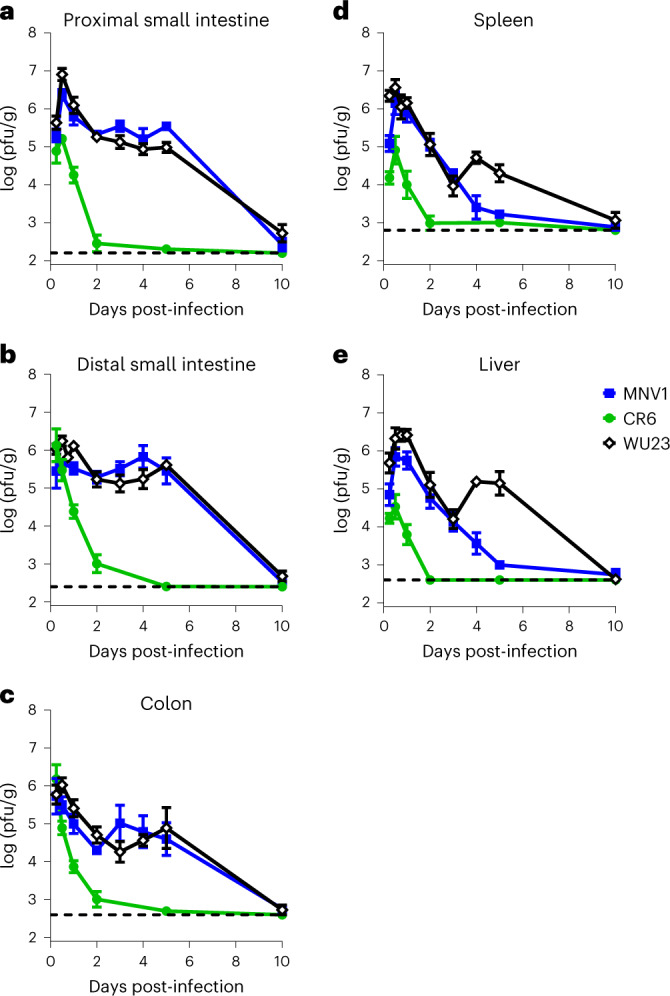
WU23 and MNV1 infect intestinal and extraintestinal tissues of neonatal B6 mice. Groups of P3 C57BL/6J (B6) pups (*n* = 5 per condition) were infected by i.g. with 10^7^ TCID_50_ units of MNV1, WU23, CR6 or mock inoculum. Virus titers were determined by plaque assay for the duodenum (**a**), ileum (**b**), colon (**c**), spleen (**d**) and liver (**e**) at the indicated timepoints. Mice from a minimum of two different litters per condition were analyzed. Data are presented as mean ± s.e.m.; dashed line indicates limit of detection.

To thoroughly characterize the course of infection in B6 neonates, P3 B6 pups were infected by i.g. with 10^7^ TCID_50_ units MNV1, CR6 or WU23 or mock inoculum and virus titers determined in intestinal and extraintestinal tissues over a 10 day time period. MNV1 and WU23 titers peaked at 12 h post-infection (hpi) in all tissues analyzed and were cleared by 10 dpi, and titers were generally comparable between the two viruses (Fig. 4). One notable difference was that WU23 titers in extraintestinal tissues were higher than MNV1 titers at 4–5 dpi (Fig. 4d,e), consistent with higher extraintestinal titers in adult B6 mice (Fig. 1d,e). CR6 titers also peaked at 6–12 hpi, depending on the tissue, but there were several notable differences compared with the more virulent MNV1 and WU23 viruses. First, peak CR6 titers in the duodenum (Fig. 4a), spleen (Fig. 4d) and liver (Fig. 4e) were 1–2 logs lower than peak MNV1 or WU23 titers. Second, CR6 clearance from all tissues occurred more rapidly than MNV1 or WU23 clearance. This latter point is surprising since CR6 establishes persistence in adult B6 mice while MNV1 is cleared acutely^10^. However, it is important to note that tuft cells, the CR6 persistence reservoir, are not present in mice this young (Kennedy et al., unpublished data).

**Fig. 5:**
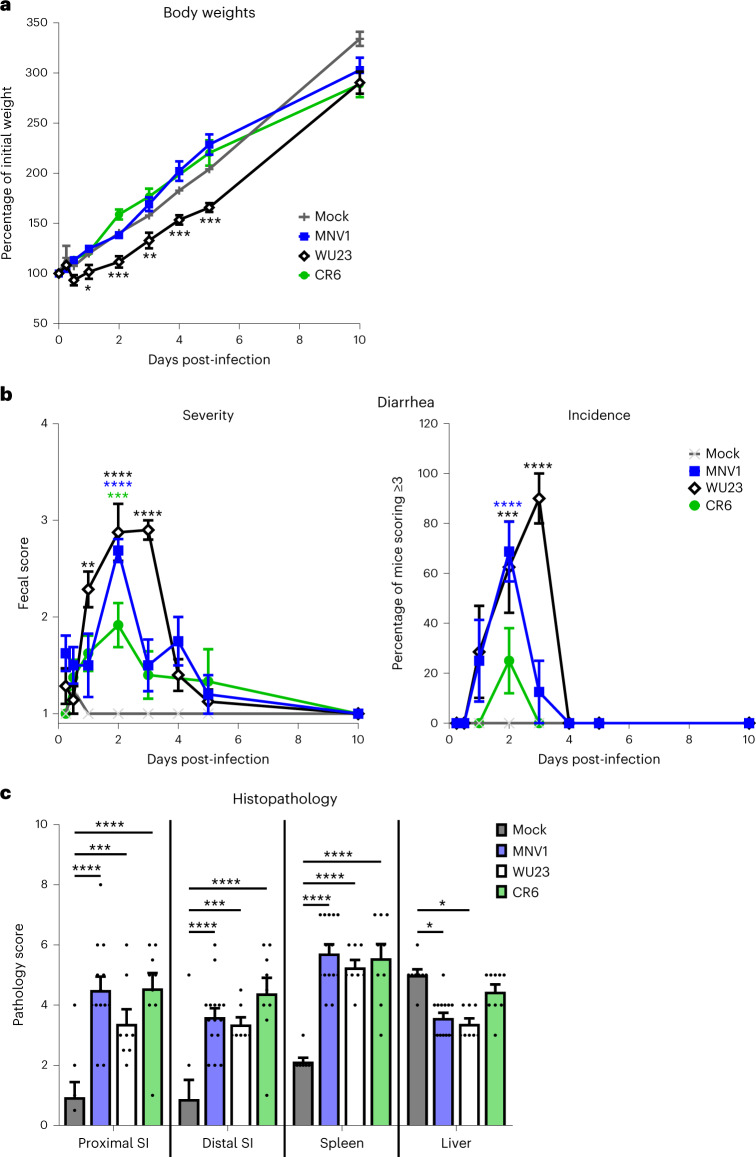
WU23 causes a transient reduction in weight gain and severe diarrhea, but modest histopathology in neonatal B6 mice. **a**, Groups of P3 C57BL/6J (B6) pups (*n* = 10–20 per condition) were infected by i.g. with 10^7^ TCID_50_ units of MNV1, WU23, CR6 or mock inoculum and were weighed at the indicated timepoints. Weights were compared with 0 dpi to determine the percent of initial weight. **b**, Fecal consistency was determined at each timepoint by palpating the pups’ abdomens. Mice that did not defecate were excluded from analysis. The proportion of mice scoring a 3 or above is presented as incidence of diarrhea. Data are presented as mean ± s.e.m.; *P* values were determined using two-sided ANOVA with corrections for multiple comparisons using Tukey’s test. Significance is reported against mock infection with black asterisks indicating WU23, blue asterisks indicating MNV1 and green asterisks representing CR6. **c**, Groups of P3 B6 pups were infected by i.g. with 10^7^ TCID_50_ units of MNV1, WU23 or mock inoculum. At 2 dpi, tissue sections were collected and stained with hematoxylin and eosin. Sections were scored blindly for pathological changes in the indicated segments of the small intestine (SI), spleen and liver. At least seven mice from at least two independent litters were analyzed for each condition. *P* values were determined using two-sided ANOVA with corrections for multiple comparisons using Tukey’s test. *P < 0.05, **P < 0.01, ***P < 0.001 and ****P < 0.0001.

We also monitored for disease in these pups, recording body weights and fecal consistency scores. While neonates infected with MNV1 and CR6 gained weight at similar rates to mock-inoculated mice, pups infected with WU23 gained 10–15% less weight 1–5 dpi (Fig. 5a). Their weights rebounded to control levels by 10 dpi. Consistent with our previous findings^22^, MNV1 caused diarrhea at 2 dpi that resolved by 3 dpi, while CR6 was attenuated (Fig. 5b). WU23-induced diarrhea was of comparable severity to MNV1 but was prolonged, beginning at 1 dpi and peaking at 3 dpi, with resolution by 4 dpi (Fig. 5b). The incidence of WU23-induced diarrhea reached 82% at 3 dpi compared with peak incidence of 61% and 18% for MNV1 and CR6, respectively. On the basis of the absence of tuft cells in neonatal mice, it was surprising to observe any disease in CR6-infected pups. Infection with 10^8^ TCID_50_ units CR6 (tenfold more virus than used in other experiments) failed to cause increased disease (Supplementary Fig. 2), suggesting that this modest diarrhea results from a host response to the virus inoculum in contrast to low-level tuft cell-independent CR6 replication.

To further characterize disease severity in MNV-infected B6 neonates, we next analyzed histopathological changes (Fig. 5c). Mirroring findings in human studies^17,31–34^ and our previous findings with MNV1- and CR6-infected neonates^22^, mice infected with MNV1, CR6 or WU23 showed modest intestinal pathological changes compared with mock-inoculated mice that included crypt hyperplasia, epithelial vacuolation and lacteal dilation (Supplementary Fig. 3a). There was no correlation between the severity of these histopathological changes and diarrhea since all three viruses caused comparably mild pathology (Fig. 5c). Likewise, all three viruses caused modest and comparable splenic pathology (Fig. 5c and Supplementary Fig. 3b). Surprisingly, diarrheagenic MNV1 and WU23 were scored as having reduced liver pathology compared with mock- and CR6-infected pups, reflecting reductions in both extramedullary hematopoiesis and vacuolation (Fig. 5c and Supplementary Fig. 3c). Given the negative correlation between diarrhea and liver pathology, the liver sections were scored independently by a second pathologist to increase confidence in these results. Again, there was significantly less vacuolation in MNV1- and WU23-infected pups compared with mock- and CR6-infected pups. Thus, MNV diarrhea is associated with reduced vacuolation in the liver, possibly indicating a malabsorptive state that causes a compensatory release of lipid stores. Overall, these results demonstrate that WU23 induces severe but self-resolving diarrhea, reflecting the pathology observed during human norovirus infection. They also illuminate a clear virus strain-dependent virulence profile in neonatal B6 mice: WU23 > MNV1 > CR6. Finally, they reveal a surprising correlation between MNV diarrhea and extraintestinal infection.

### Diarrheagenic MNV strains infect subepithelial cells in vivo

**Fig. 6:**
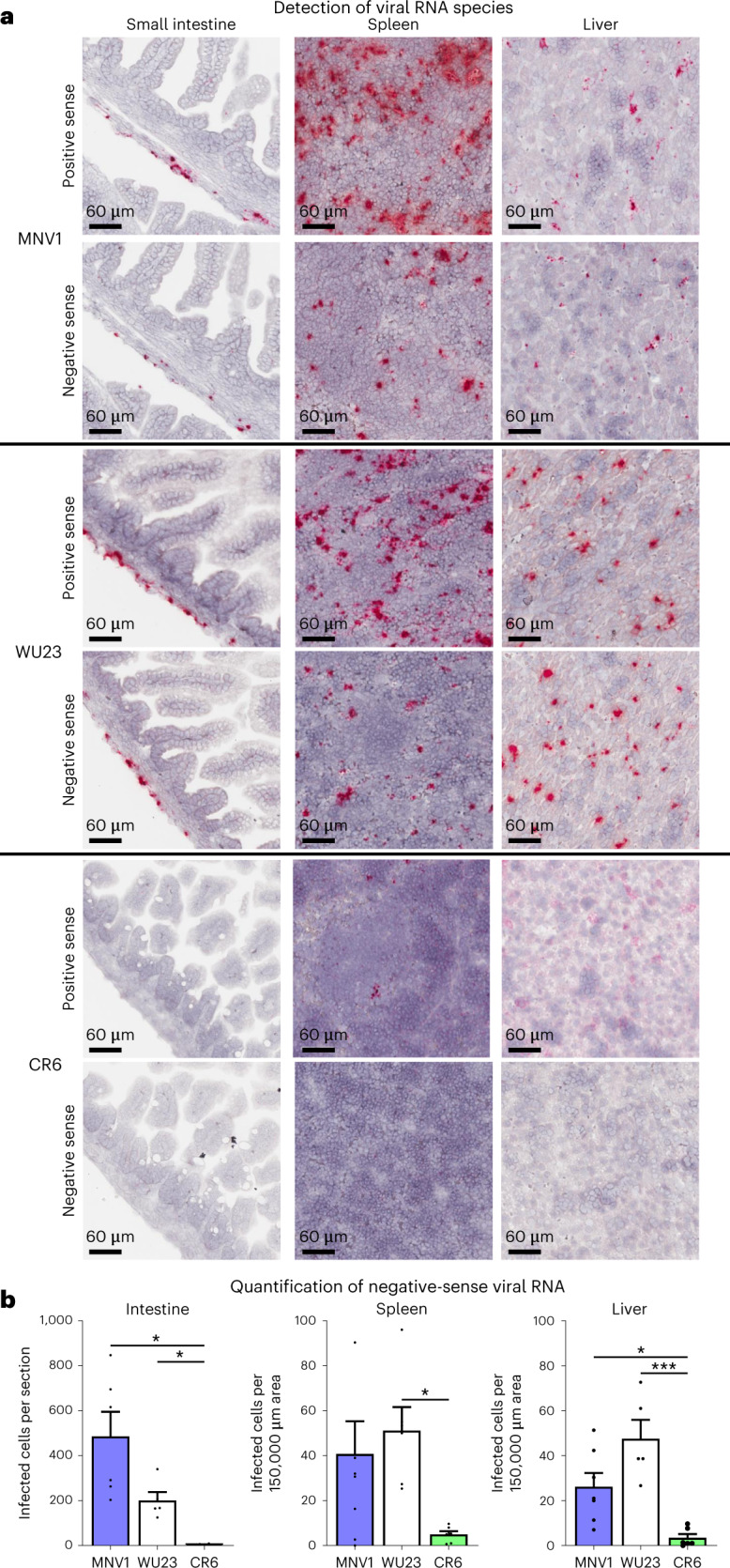
Virulence correlates with infection of subepithelial cells in the intestine and extraintestinal tissues. **a**, Groups of P3 C57BL/6J (B6) neonates were infected by i.g. with 10^8^ TCID_50_ units of MNV1, CR6 or WU23 or mock inoculum. Tissue sections from the small intestine, spleen and liver collected from pups at 18 hpi were probed for viral positive-sense and negative-sense RNA. Tissue sections from at least four mice collected from a minimum of two different litters per condition were analyzed and representative images are shown. **b**, Negative-sense viral RNA was quantified from the entire intestinal section of each mouse. For spleen and liver sections, due to variable tissue sizes on the slides, we randomly selected five areas per section of 150,000 μm^2^ each and reported the average of these values for each mouse. Data are presented as mean ± s.e.m; *P* values were determined using one-sided ANOVA with corrections for multiple comparisons using Tukey’s test. *P < 0.05, **P < 0.01, ***P < 0.001 and ****P < 0.0001.

To confirm the virus strain-dependent differences in tropism, in situ hybridization (ISH) was performed on tissue sections from infected pups using probes to the positive-sense viral genomes (which detects both infected cells and free virus) and negative-sense viral RNA species (produced only in infected cells). Positive-sense viral RNA was detected in the epithelium of the most distal region of the small intestine for all three viruses but was not accompanied by any viral replication (Supplementary Fig. 4). This pattern is consistent with nonspecific uptake of virus into ileal enterocytes in a neonatal-specific process, which aligns with recent work by Kennedy et al. (unpublished data). It should be noted that this pattern does not correlate with virulence since it was observed for all three viruses to comparable levels, nor does it result in viral replication inside the cells. On the other hand, diarrheagenic MNV1 and WU23 replicated in the submucosa of the small intestine, as indicated by the detection of abundant negative-sense viral RNA (Fig. 6a,b). This did correlate with disease since no CR6 submucosal replication was detected. It was unexpected and intriguing that both diarrheagenic viruses replicated only in submucosal cells, demonstrating a potential link between cell-associated dissemination and intestinal disease. Consistent with this and with viral titers, MNV1 and WU23 were detected at much higher levels in the spleen and liver than CR6 (Fig. 6a,b). Both diarrheagenic viruses replicated in extraintestinal tissues, as evidenced by detection of negative-sense viral RNA. Overall, these findings are consistent with prior work demonstrating MNV1 infection of immune cells but not epithelial cells^14,22^. They extend previous findings by revealing (1) a similar tropism of a diarrheagenic MNV isolated from an enteric site for subepithelial cells; and (2) virus replication in extraintestinal tissues for both diarrheagenic viruses but not an attenuated virus.

**Fig. 7:**
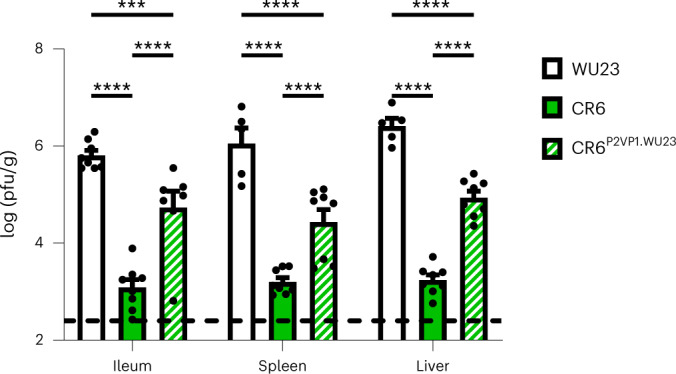
The hypervariable P2 domain of VP1 controls dissemination in neonates. Groups of P3 C57BL/6J (B6) neonates (*n* = 8 per condition) were infected by i.g., with 10^8^ TCID_50_ units of WU23, CR6 or the chimeric virus CR6^P2VP1.WU23^ and tissues collected at 2 dpi. Virus titers were determined by plaque assay. Mice from a minimum of two different litters per condition were analyzed. Data are presented as mean ± s.e.m.; dashed line indicates limit of detection. *P* values were determined using two-sided ANOVA with corrections for multiple comparisons using Tukey’s test. *P < 0.05, **P < 0.01, ***P < 0.001 and ****P < 0.0001.

We previously reported that the hypervariable protruding 2 (P2) domain of the VP1 capsid protein determines diarrheagenic potential of MNV in neonatal mice^35^. The VP1 P domain also regulates MNV dissemination in adult *Stat1*^−*/*−^ mice^27^. Thus, it was logical to predict that the VP1 P2 domain likewise determines dissemination in neonates. To test this, we infected P3 mice with parental WU23, parental CR6 or chimeric CR6^P2VP1.WU23^ and analyzed viral titers at 48 hpi. As predicted, CR6^P2VP1.WU23^ spleen and liver titers were significantly higher than CR6 titers (Fig. 7). These results further strengthen the correlation between norovirus intestinal disease and dissemination.

### Type I IFNs protect from WU23 diarrhea while type III IFNs exacerbate disease

**Fig. 8:**
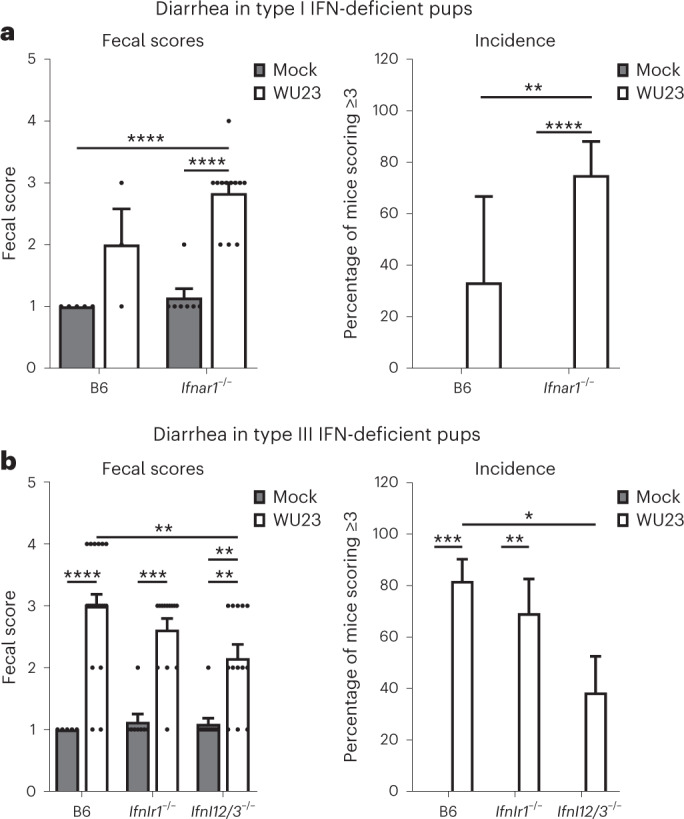
Type I IFNs protect against MNV diarrhea in neonates while type III IFNs exacerbate disease. **a**, Groups of P3 C57BL/6J (B6) or B6-*Ifnar1*^−*/*−^ pups (*n* = 7–18 per condition) were infected by i.g. with 1 TCID_50_ unit of WU23 or mock inoculum. Left: at 2 dpi, fecal consistency was determined by palpating their abdomens. Mice that did not defecate were excluded from analysis. Right: the proportion of mice scoring a 3 or above is presented as incidence of diarrhea. **b**, Groups of P3 B6, *Ifnlr*^−*/*−^ and *Ifnl2/3*^−*/*−^ pups (*n* = 16–25 per condition) were infected by i.g. with 10^7^ TCID_50_ units of WU23 or mock inoculum. Left: at 2 dpi, fecal consistency was determined by palpating their abdomens. Mice that did not defecate were excluded from analysis. Right: the proportion of mice scoring a 3 or above is presented as incidence of diarrhea. Mice from at least two independent litters per condition were analyzed. Data are presented as mean ± s.e.m.; *P* values were determined using two-sided ANOVA with corrections for multiple comparisons using Tukey’s test. *P < 0.05, **P < 0.01, ***P < 0.001 and ****P < 0.0001.

It has been well established that type III IFNs provide protection from enteric viruses that infect epithelial cells (for example, rotavirus and CR6) whereas type I IFNs protect from systemic spread. Our results revealing that diarrheagenic MNV strains infect subepithelial cells but not intestinal epithelial cells compelled us to test the role of type I and type III IFNs in protecting from MNV diarrhea. We first infected P3 *Ifnar1*^−*/*−^ mice with 10^7^ TCID_50_ units WU23, the dose used in B6 infections, but 70% of the pups succumbed to infection by 2 dpi (Supplementary Fig. 5a). Because of this, we were unable to profile diarrhea severity at this dose. Likewise, 100% of pups infected with 10^6^ TCID_50_ units WU23 and 60% of pups infected with 10^4^ TCID_50_ units succumbed to infection (Supplementary Fig. 5a). While the majority of neonates survived infection with doses lower than 10^4^ TCID_50_ units WU23, animals infected with a dose as low as 10 TCID_50_ units were visibly moribund and not suckling. On the other hand, pups infected with 1 TCID_50_ unit appeared healthy and were suckling. Using this dose, the severity and incidence of diarrhea in WU23-infected *Ifnar1*^−*/*−^ was significantly higher than in wild-type pups infected with the same dose (Fig. 8a). Importantly, viral titers were detected in B6 mice infected with 1 TCID_50_ unit WU23, demonstrating that this low dose does infect neonatal mice (Supplementary Fig. 5b). We also assessed the role of type III IFN in protecting from WU23-induced diarrhea by infecting P3 *Ifnlr1*^−*/*−^ and *Ifnl2/3*^−*/*−^ neonates with 10^7^ TCID_50_ units WU23. Unexpectedly, *Ifnl2/3*^−*/*−^ pups developed significantly reduced diarrhea compared with B6 controls (Fig. 8b). There was a trend towards reduced disease in *Ifnlr1*^−*/*−^ pups as well, although the difference was not statistically significant. These findings reveal that type III IFN actually contributes to norovirus diarrhea.

**Fig. 9:**
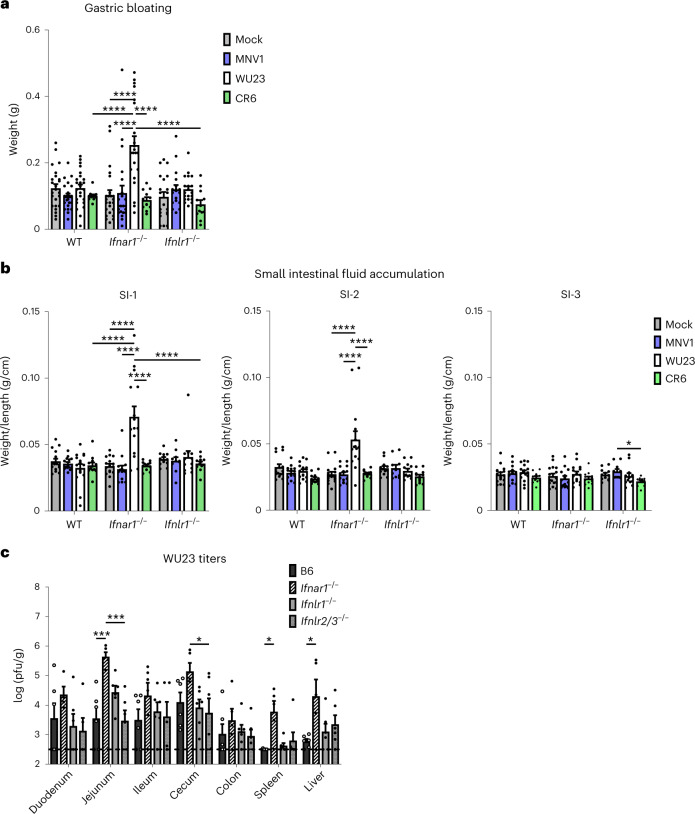
Type I IFNs protect against WU23 intestinal disease in adult mice. Groups of adult C57BL/6J (B6), *Ifnar*^−*/*−^ and *Ifnlr*^−*/*−^ mice (*n* = 12–25 per condition) were p.o. infected with 10^7^ TCID_50_ units of MNV1, WU23, CR6 or mock inoculum and killed at 3 dpi. **a**, Stomachs were ligated, weighed, contents removed and then weighed again to calculate weight of stomach contents. **b**, To calculate small intestinal fluid accumulation, intestines were ligated into thirds. Each section was weighed and length measured to determine the weight-to-length ratios. Mice from three independent experiments were analyzed for all conditions. WT, wild type. **c**, Groups of adult mice of the indicated strains (*n* = 6 per condition) were p.o. infected with 10^7^ TCID_50_ units of WU23. At 1 dpi, virus titers were determined in the indicated segments of the intestinal tract, spleen or liver by plaque assay. Data are presented as mean ± s.e.m.; *P* values were determined using two-sided ANOVA with corrections for multiple comparisons using Tukey’s test. Dashed line indicates limit of detection. *P < 0.05, **P < 0.01, ***P < 0.001 and ****P < 0.0001.

To determine whether the importance of type I IFN was age specific, we also examined virulence in adult knockout mice. Several assays were used to quantify intestinal disease at 3 dpi, the peak of intestinal disease in MNV1-infected *Stat1*^−*/*−^ mice^26,36^. First, stomach contents were weighed since gastroparesis has been noted in people infected with human noroviruses^37^ and in *Stat1*^−*/*−^ mice infected with MNV1 (ref. ^26^). Second, small intestinal weights were normalized to length as a quantitative measurement of fluid accumulation in the gut lumen. There were no significant differences in these indicators of intestinal disease in adult wild-type B6 mice infected with 10^7^ TCID_50_ units MNV1, WU23 or CR6 compared with mock-inoculated mice (Fig. 9a,b). To investigate the role of type I and type III IFNs in controlling MNV intestinal disease, we also infected *Ifnar1*^−*/*−^ and *Ifnlr1*^−*/*−^ mice with each virus. While neither mouse strain developed intestinal disease upon MNV1 or CR6 infection, *Ifnar1*^−*/*−^ mice infected with WU23 displayed significant gastric bloating (Fig. 9a), small intestinal fluid accumulation (Fig. 9b) and liquid stools (Supplementary Fig. 6). Interestingly, fluid accumulation was observed in the proximal two-thirds of the small intestine but not in the distal third (Fig. 9b). In contrast to results in *Ifnar1*^−*/*−^ mice, WU23-infected *Ifnlr1*^−*/*−^ mice did not develop gastric bloating, intestinal fluid accumulation or diarrhea (Fig. 9a,b). Consistent with increased disease in WU23-infected *Ifnar1*^−*/*−^ adult mice, WU23 titers were generally higher in this strain than in wild-type or type III IFN-deficient mice (Fig. 9c). Overall, these results demonstrate that WU23 is more virulent than MNV1 or CR6 since it caused intestinal disease in *Ifnar1*^−*/*−^ mice while MNV1 and CR6 did not. Furthermore, they reveal that type I IFNs protect neonatal and adult mice from MNV-induced intestinal disease whereas type III IFNs are dispensable in adults and pathologic in neonates. These findings further corroborate the importance of immune cell infection in the development of norovirus diarrhea.

## Discussion

Noroviruses are a major cause of gastroenteritis across the globe, causing a remarkable 685 million cases per year, yet limitations in model systems have hindered the field’s ability to elucidate disease mechanisms. The development of the MNV model nearly two decades ago has facilitated progress in understanding host–norovirus interactions and norovirus strain variability. However, it is not regarded as a strong model of norovirus gastroenteritis because MNV strains were either isolated from extraintestinal tissue, raising concerns about translatability of research findings to human norovirus disease (for example, MNV1), or they do not cause intestinal disease (for example, CR6). Consequently, our understanding of norovirus pathogenesis remains poorly defined today. Herein we provide a detailed characterization of a diarrheagenic MNV strain called WU23, originally isolated from feces of a mouse experiencing diarrhea. WU23 uses the same host receptor CD300lf as prototype MNV strains^11^ but causes more severe intestinal disease in genetically wild-type neonates and type I IFN-deficient adult mice. This novel model system shares all the hallmark features of human norovirus infection, thus representing a biologically relevant model to probe norovirus disease mechanisms.

We used this superior model system to make two key discoveries about norovirus pathogenesis. First, diarrheagenic MNV strains commonly infect subepithelial cells likely to be immune cells based on the established MNV1 tropism in vitro and in adult mice^14,22,38,39^, but not epithelial cells in the intestine. While the paradigm in the field is that human noroviruses infect intestinal epithelial cells and not immune cells, collective data in the literature argue that there is marked variability in norovirus cell tropism even among highly genetically related strains. For example, MNV1 infects intestinal immune cells in adult mice^14^ while MNV-CR6 infects tuft cells^15^ although their VP1 proteins share 94% identity. Biopsies of human norovirus-infected people likewise demonstrate variable cell tropism: one biopsy study identified intestinal epithelial cells as the major viral target in an immunocompromised patient^21^, while another study detected viral antigen predominantly in immune cells in a cohort of immunocompromised patients^17^. Shown in this latter study were both structural and nonstructural viral proteins in lamina propria macrophages^17^. While Karandikar et al. concluded that epithelial cells are the main viral target in their study despite the majority of viral antigen being detected in lamina propria immune cells, their argument for why their results do not reflect immune cell infection—that lamina propria macrophages acquire virus antigens via phagocytosing infected epithelial cells—is flawed for several reasons. First, while it is true that lamina propria macrophages phagocytose material, they do so even under homeostatic conditions as highlighted by the detection of comparable amounts of an epithelial marker in this cell type in mock and infected tissue^17^. Thus, detection of an epithelial marker in virus-positive macrophages does not prove that intracellular viral antigen was acquired by phagocytosis. It could also have been acquired upon viral replication inside the cell. Second, there was more abundant viral antigen in the lamina propria than in the epithelium. In fact, intestinal epithelial cells are nonprofessional phagocytes, so one could make a similar argument that they have simply phagocytosed infected immune cells in the vicinity. Overall, detection of viral nonstructural protein or negative-sense viral RNA is the standard approach for identifying cells infected by positive-sense RNA viruses. Applying this standard, Karandikar et al. demonstrated that intestinal immune cells are the predominant target cells in these biopsies while epithelial cells are minor targets^17^. It should be noted that human norovirus strains are much more genetically diverse than MNV strains. For example, two intra-cluster GII.4 strains can have as many as 60 amino acid differences in their VP1 proteins, sharing 89% identity^40^. On the basis of existing data, it is thus entirely plausible, even probable, that there is strain-dependent variability in human norovirus cell tropism. With this in mind coupled with the small sample sizes of biopsies tested for human norovirus cell tropism and the immunocompromised nature of these individuals that could influence cell tropism^17,21^, available data argue that human norovirus cell tropism in vivo is variable and animal models like the one described in our study are needed to link tropism to virulence. Our finding that infection of subepithelial cells is associated with diarrhea is surprising given that diarrhea generally results from dysfunction of the intestinal epithelium. It will be fascinating to interrogate how noroviruses can cause diarrhea in the absence of intestinal epithelial cell infection, with a potential contribution from immunopathological effects of underlying intestinal immune cell infection.

Consistent with norovirus infection of subepithelial cells, our findings revealed that type I IFNs are critical to protect from intestinal disease in both adult and neonatal hosts whereas type III IFNs are dispensable in adults and, surprisingly, exacerbate diarrhea in neonates. While it has long been known that IFN responses are important for protecting from MNV infections in adult mice^36,41^, there are conflicting data in the literature on the importance of type I versus type III IFNs in the MNV model system. Type I IFNs prevent severe weight loss and lethality in MNV1-infected mice^42,43^, while type III IFNs are important in controlling persistent colonic infection by the MNV strain CR6 (ref. ^44^), leading to the current paradigm that type III IFNs provide protection against intestinal MNV infection while type I IFNs are critical to prevent systemic infection^45,46^. Yet no study examining MNV infection in *Ifnar1*^−*/*−^ or *Ifnlr1*^−*/*−^ mice measured intestinal disease, using instead nonspecific virulence measures of weight loss and lethality or virus titers to assess the role of specific classes of IFNs. Our studies herein unequivocally demonstrate a dominant role for type I IFNs in protecting from MNV-induced intestinal disease. This finding is consistent with a human norovirus study that demonstrated that IFNAR1 protects from human norovirus replication in a virus-strain-dependent manner while IFNLR1 is dispensable^47^. The observation that WU23-induced diarrhea was less severe in *Ifnl2/3*^−*/*−^ neonates was unexpected for two reasons. First, type III IFNs have well-established antiviral activity in other infections^48^, but our results instead suggest that they exacerbate norovirus diarrhea. Second, diarrheagenic MNV strains were not detected in intestinal epithelial cells, which are the main site of type III IFN receptor expression. Yet our results are in line with an increasing appreciation for the capacity of type III IFNs to exacerbate disease associated with viral infections in situations where immune cell-derived type III IFNs reduce tissue repair^49–51^, raising the intriguing possibility that norovirus infection of intestinal immune cells leads to production of type III IFNs that impair epithelial barrier function, contributing to diarrhea, while type I IFNs protect from disease.

The second key discovery made using the WU23 model system is a positive association between norovirus intestinal disease and dissemination to extraintestinal tissues. Consistent with dissemination as a common feature of norovirus infection, human norovirus genome or antigen has been detected at extraintestinal tissues in Yucatan miniature piglets^52^ and in the blood of children^53–56^, immunocompromised adults^57^ and numerous animal models^7,19,20,52,58^. The infection of the liver by two diarrheagenic MNV strains is intriguing given the extensive crosstalk between the liver and the gut. Dissemination to the liver has also been reported for human noroviruses in chimpanzees and zebrafish^19,58^, suggesting this is a common feature among enteric noroviruses. It is possible that liver infection results in dysregulated metabolic activity that contributes to diarrhea, supported by our finding that both diarrheagenic MNV strains cause a reduction in lipid droplets in the liver. This new small animal model will enable testing of this hypothesis. While other exciting new model systems have been reported for studying human noroviruses in recent years, they have limitations in interrogating mechanisms of disease. For example, human noroviruses can infect B cells and enteric organoids in vitro^39,59^ but these systems inherently lack the complexity of multicellular, multilayered tissue structures present within a host. Zebrafish larvae support human norovirus replication and virus disseminates to the liver in this model; however, this is an asymptomatic model of infection^58^. Thus, MNV infection of their natural hosts represents a powerful platform to elucidate pathophysiological processes leading to norovirus diarrhea.

As was previously reported for MNV1, host susceptibility to WU23 diarrhea is tightly age restricted. This has also been reported for rotavirus infection of mice that has been linked to physiological and immunological maturation of the intestine, although in this case resistance to disease correlates with resistance to infection^60–62^. Our finding of age restriction to MNV-induced diarrhea but not infection suggests a distinct underlying mechanism. Although we do not currently have an explanation for this observation, one possibility is that the virus targets distinct types of immune cells in diarrhea-susceptible versus diarrhea-resistant mice. MNV1 can infect macrophages, dendritic cells, B cells, T cells and neutrophils in adult mice^14,39^, and the numbers and functionality of these cell types in the gut change during development. Our previous findings suggest that infection of lymphocytes plays a key role in disease^22,35^, so it is possible that there are age-regulated differences in the susceptibility of B or T cells to MNV infection or an age-regulated difference in the response of these cells to infection. This area of research will be fascinating to explore in future studies. Overall, this body of work illustrates the importance of translatable small animal models for understanding basic norovirus biology to elucidate disease mechanisms.

## Methods

### Cells and viruses

BV2 and HEK293T cells were maintained in Dulbecco’s modified Eagle medium supplemented with 10% fetal bovine serum, 100 U penicillin/ml and 100 μg/ml streptomycin. Cells tested negative for mycoplasma. MNV-1.CW3 (GenBank accession number KC782764, referred to as MNV1) and MNV-CR6 (GenBank accession number JQ237823.1, referred to as CR6) infectious clones were used to generate virus stocks. Because a WU23 infectious clone was not available, the WU23 sequence (GenBank accession number EU004668.1) was synthesized by Genscript and cloned into pSP73. A minimal CMV promoter was included at the 5′, end and a poly(A) tail followed by a ribozyme sequence was included at the 3′ end. Two nucleotides labeled as ‘Y’ in the GenBank sequence were synthesized as thymine residues based on sequencing the original viral stock. This clone was replication incompetent. Comparing the sequence of our original WU23 stock to the GenBank sequence, we noted that residues 4,826 and 4,828 were different, so we introduced the mutations A4826C and G4828A using the Agilent QuikChange Lightning site-directed mutagenesis kit. This WU23 clone was infectious. To generate virus stocks, 10^6^ HEK293T cells were transfected with 5 µg of endotoxin-free infectious clone using Lipofectamine 3000. Cells were frozen after 30 h and lysed by freeze–thaw, and lysates were applied to BV2 cells. BV2 lysates were frozen when cultures displayed 90% cytopathic effect and then freeze–thawed twice, and cell debris was removed by low-speed centrifugation, followed by purification of virus through a 25% sucrose cushion. Virus was dissolved in phosphate-buffered saline (PBS). Virus stocks were titered by a standard TCID_50_ assay as described previously^10^. Stocks were sequenced to confirm no mutations arose during generation. Initial WU23 experiments were performed with original virus (kindly provided by Dr. Craig Wilen, Yale University). Once the infectious clone plasmid was available, virus stock for WU23 was made up in the same way as other stocks. Both methods of producing WU23 stock produced similar in vivo results (Supplementary Fig. 7). A mock inoculum stock was prepared in the same manner using BV2 lysate from uninfected cultures.

### Mice

Specific pathogen-free mice used in this study were bred and housed in animal facilities at the University of Florida and Washington University School of Medicine. All animal experiments were performed in strict accordance with federal and university guidelines and approved by the Institutional Animal Care and Use Committee at the University of Florida (study numbers 20190632, 201810473, 202110473 and 202200000065). The conditions in animal rooms used in this study fall within the standards set by the ‘Guide for the Care and Use of Laboratory Animals’. Mice are housed in Nexgen cages with external water bottles containing autoclaved water, and the cages and water are changed weekly. Mice are fed an Envigo 7012 autoclaved diet. They are housed in a room on a 14 h light–dark cycle (5:00 to 19:00) Additionally, all mice were bred under MNV-free conditions.

Age- and sex-matched 6–12-week-old C57BL/6J (Jackson no. 000664, referred to as B6), C57BL/6J-*Ifnar1*^−*/*−^ (Jackson no. 010830, referred to as *Ifnar1*^−*/*−^) and C57BL/6J-*Ifnlr1*^−*/*−^ (referred to as *Ifnlr1*^−*/*−^)^63,64^, mice were used in adult mouse infections. Adult mice were inoculated perorally (p.o.) with 10^7^ TCID_50_ units of the indicated virus strain or mock inoculum. For adult virulence studies, mice were killed and intestines and stomachs collected at 3 dpi. For adult survival and weight kinetics, mice were weighed on day 0 and every 24 h thereafter for 7 days. For measuring gastric contents, the esophageal and intestinal junctions of the stomach were ligated, dissected and weighed. The stomachs were then opened, contents removed and reweighed. For measuring intestinal fluid, three equal sections from the pyloric to the cecal junctions were ligated, dissected and weighed. The lengths were also measured. Small intestinal fluid accumulation was determined by dividing the weight by the length of each section.

For neonatal mouse experiments, male and female BALB/c (Charles River no. 028), B6, *Ifnar1*^−*/*−^, *Ifnlr1*^−*/*−^ and C57BL/6J-*Ifnl2/3*^−*/*−^ (ref. ^64^) (referred to as *Ifnl2/3*^−*/*−^) pups were used. For all neonatal mouse experiments except for age-restriction experiments, 3-day-old neonatal mice were inoculated with the indicated MNV strain or mock inoculum using o.g.^22^ or i.g. For the latter procedure, 40 μl of inoculum was injected directly into the stomach (which can be visualized through the skin in the upper left quadrant) using a 30-gauge needle and 1 ml BD syringe. For neonatal virulence studies, pups were weighed at the indicated timepoints, and the abdomen of each pup was palpated to induce defecation. Fecal condition was assessed unblinded on the basis of color and consistency according to a 6-point scale: 0, no defecation; 1, firm, orange, does not smear; 2, pasty, orange or mixed color, does not smear; 3, orange or yellow, semi-liquid and smears; 4, yellow, liquid and smears; 5, green-yellow, nonviscous liquid. Any pup that scored a 3–5 was considered to have diarrhea for the purpose of calculating incidence.

### Virus titration assays

For virus stock production, virus titers were determined by a standard TCID_50_ assay as described previously^10^ with minor modifications. Eight replicates each of multiple dilutions per sample were applied to BV2 cells. Cytopathic effect was scored at 3 dpi. For virus titer determination in animal tissues, plaque assays were performed. Collected tissues were immediately placed in tubes with 1.0 mm zirconia/silica beads and 1 ml of complete Dulbecco’s modified Eagle medium and flash frozen. Samples were subsequently thawed and homogenized by bead beating. Multiple dilutions of tissue samples were applied to BV2 cells. Plates were then incubated at room temperature with rocking. After 1 h, plates were overlaid with 1.5% SeaPlaque Agarose and modified Eagle medium supplemented with 10% fetal bovine serum, 100 U penicillin/ml and 100 μg/ml streptomycin. Plates were then incubated for 2 days at 37 °C. Cells were stained with 1.5% SeaKem Agarose in PBS with neutral red, and plaques counted 3–4 h later.

### Histology

Small intestinal tissue sections were collected from neonatal mice infected as described above. Tissues were rolled and fixed in 10% buffered formalin for 16 h and then transferred to PBS. Tissues were then paraffin embedded and sectioned by the University of Florida Molecular Pathology Core. Sections of 4 µm thickness were cut from each block and stained with hematoxylin and eosin by the University of Florida Molecular Pathology Core. Tissue sections were imaged using an Aperio Scanscope CS and analyzed using Leica’s Aperio ImageScope 12.4.3 slide scan software. Histology slides were scored blindly by an animal veterinarian. Intestinal sections were scored for mucosal expansion (1, acellular; 2, inflammatory), crypt depth (0, 10–20 µm; 1, 20–30 µm; 2, 30–40 µm; 3, ≥40 µm), vacuolation (0, no vacuolation; 1, vacuolation at tip or base; 2, vacuolation at tip and base) and barrier integrity, villous blunting and broadening, crypt cell hyperplasia and lacteal dilations (1, mild; 2, moderate). Spleen sections were scored for extramedullary hematopoiesis (scored 1–3), megakaryocytes (scored 1–3), disorganization (0, absent; 1, present) and vacuolation (scored 0–2). Liver sections were scored for extramedullary hematopoiesis (1, isolated sinusoids or periportal; 2, periportal aggregates, sinusoidal evident; 3, periportal aggregates, larger/distinct sinusoidal aggregates) and vacuolation (1, small intracellular; 2, larger, majority cytoplasm; 3, larger vacuoles (round) seen in addition to larger, majority cytoplasm). A clinical pathologist specializing in hepatic pathology also scored the liver sections for the presence of vacuolation (0, none or <5%; 1, 5–33%; 2, >33–66%; 3, >66%).

### RNAscope-based ISH assays

RNAScope ISH assays were performed as previously described^22^. Small intestine, spleen and liver tissue sections were fixed in 10% buffered formalin for 16 h and then transferred to PBS. Tissues were then paraffin embedded within 24 h. Sections of 4 µm thickness were then cut from each block. Tissues were deparaffinized by heating at 60 °C for 30 min followed by xylene treatment and dehydration. Sections were hybridized with custom-designed probes targeting positive-sense (ACDBio #471891, #828951) or negative-sense (ACDBio #471901 and #829991) MNV RNA for 2 h at 40 °C followed by probe amplification and detection using the ACDBio RNAScope RED detection kit. Positive (PPIB, ACDBio #313911) and negative (DapB, ACDBio #310043) control probes were stained in parallel for all experiments, and tissues from mock-inoculated mice were hybridized with viral probes as negative controls in every experiment. To visualize tissue morphology, sections were counterstained with 50% hematoxylin. Sections were imaged using a Aperio Scanscope CS and Leica’s Aperio ImageScope 12.4.3 slide scan software.

### Statistical analysis

All data were analyzed with GraphPad Prism software. *P* values were determined using one-way or two-way ANOVA with corrections for multiple comparisons. Error bars denote standard errors of mean in all figures.

### Reporting summary

Further information on research design is available in the Nature Portfolio Reporting Summary linked to this article.

## Online content

Any methods, additional references, Nature Portfolio reporting summaries, source data, extended data, supplementary information, acknowledgements, peer review information; details of author contributions and competing interests; and statements of data and code availability are available at 10.1038/s41684-023-01166-5.

## Supplementary information

Supplementary Information Supplementary Figs. 1–7.

Reporting Summary

## Data Availability

The data from this study are tabulated in the main paper and supplementary materials. All reagents are available from S.M.K. under a material transfer agreement with University of Florida.
