# Supplementary material for: Infection of neonatal mice with the murine norovirus strain WU23 is a robust model to study norovirus pathogenesis
Source: Lab Anim (NY). 2023 May 4;52(6):119–29. doi: 10.1038/s41684-023-01166-5 (PMC10234811; doi:10.1038/s41684-023-01166-5)
Supplement: Supplementary file 1 — Supplementary Figs. 1–7. [file 41684_2023_1166_MOESM1_ESM.pdf]

nature portfolio

https://doi.org/10.1038/s41684-023-01166-5

Supplementary information

Infection of neonatal mice with the murine norovirus strain WU23 is a robust model to study norovirus pathogenesis

In the format provided by the
authors and unedited

Supplemental Figure 1. WU23 does not cause overt disease in adult WT mice.

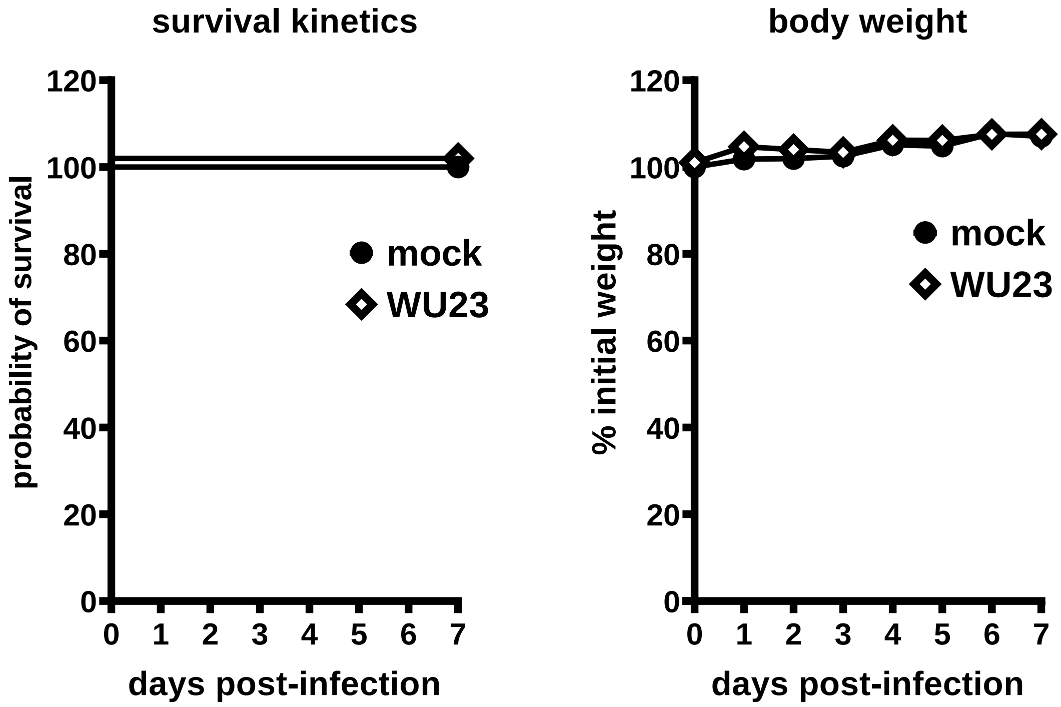

Groups of 6-week-old C57BL/6J mice (n = 5) were inoculated with 10^7 $TCID_{50}$ units of WU23 or mock inoculum. Mice were weighed on day 0 and every 24 hours thereafter for 7 days. Survival (left graph) and weight changes, calculated as the percent change compared to the initial weight (right graph) were calculated. No statistical difference was observed between the groups.

Supplemental Figure 2. High-dose CR6 infection fails to cause significant intestinal disease.

a. Diarrhea

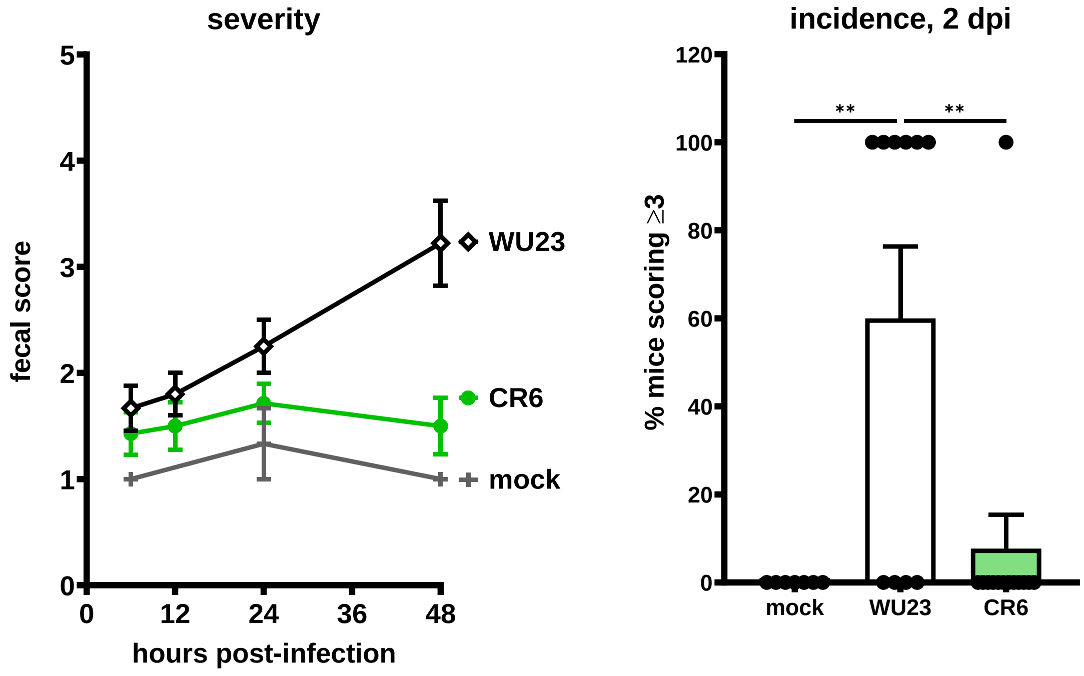

b. Colon content consistency

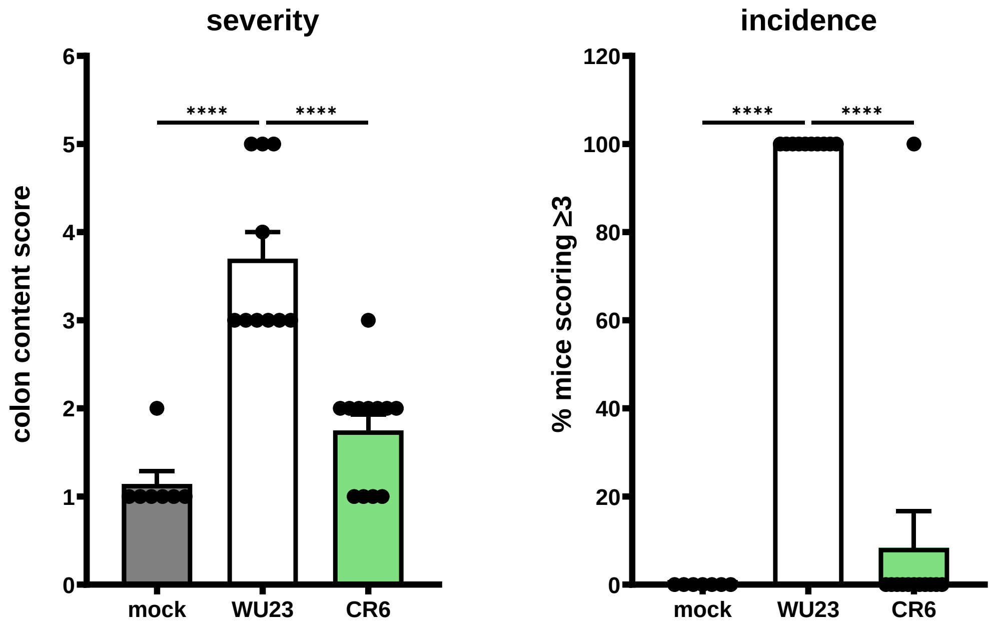

Groups of P3 B6 pups were infected by intragastric inoculation (i.g.) with 10^8 $TCID_{50}$ units of WU23, CR6, or mock inoculum. **a)** Fecal consistency was determined at each time point by palpating the pups' abdomens (left graph). Mice that did not defecate were excluded from analysis. The proportion of mice scoring a 3 or above at 2 dpi is presented as incidence of diarrhea (right graph). **b)** Colon contents were collected and scored at 2 dpi.

Supplemental Figure 3. Disease severity does not correlate with histopathology.

a. Intestinal pathology

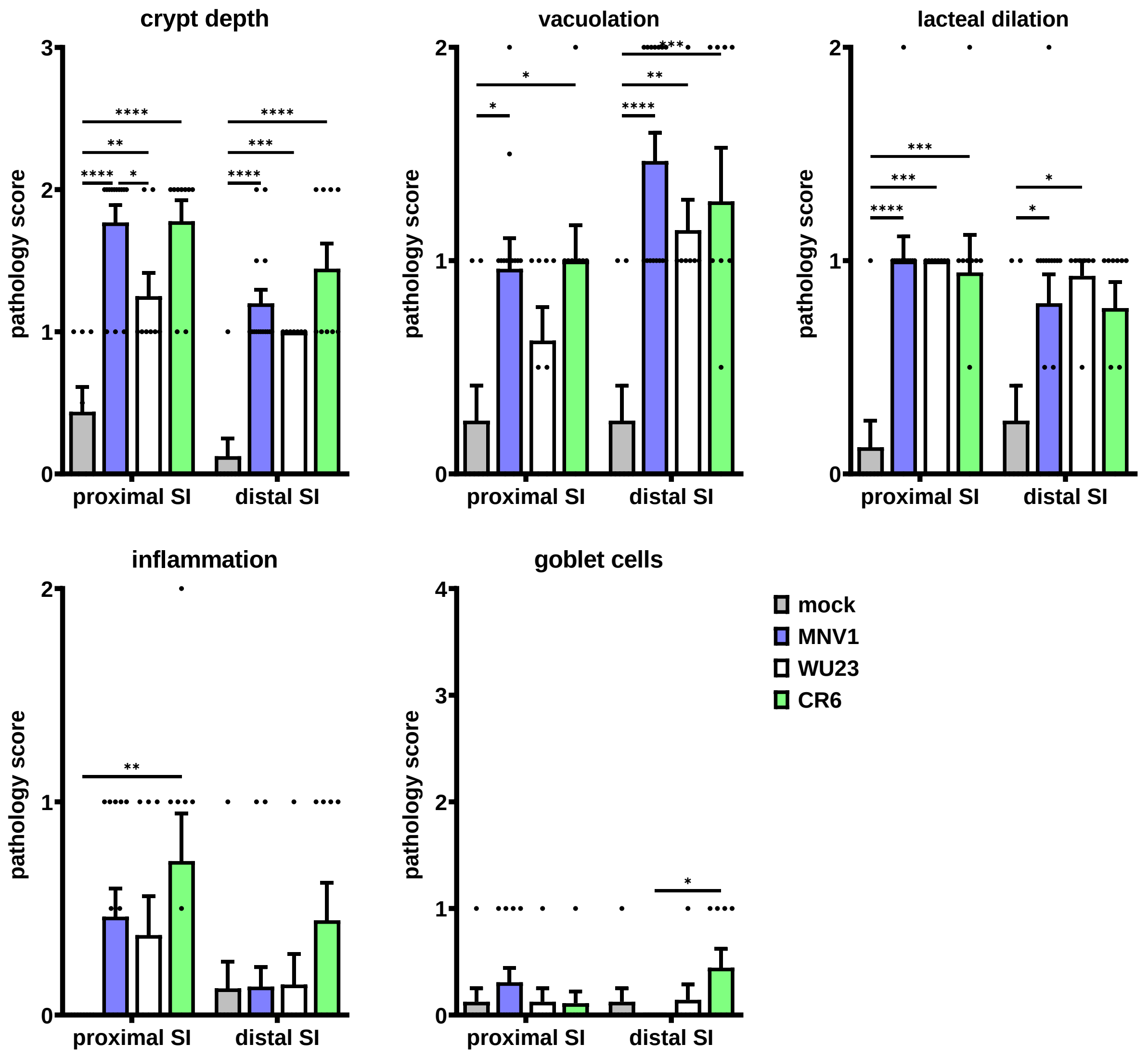

b. Spleen pathology

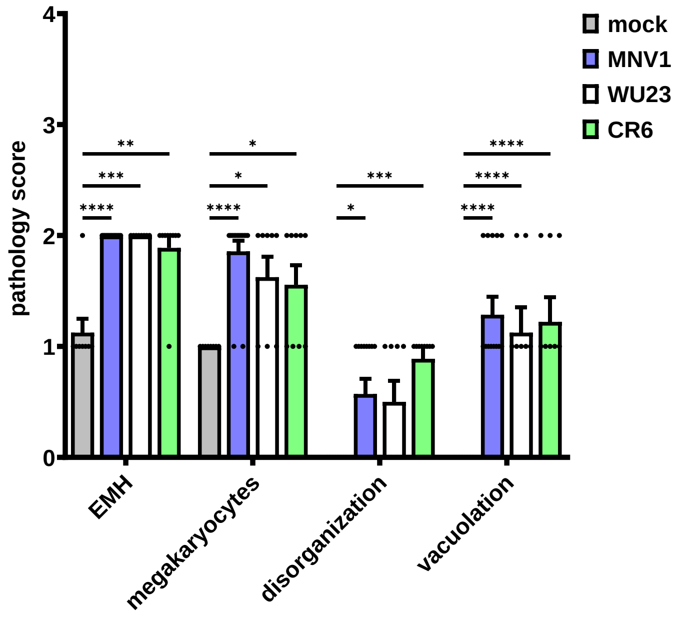

c. Liver pathology

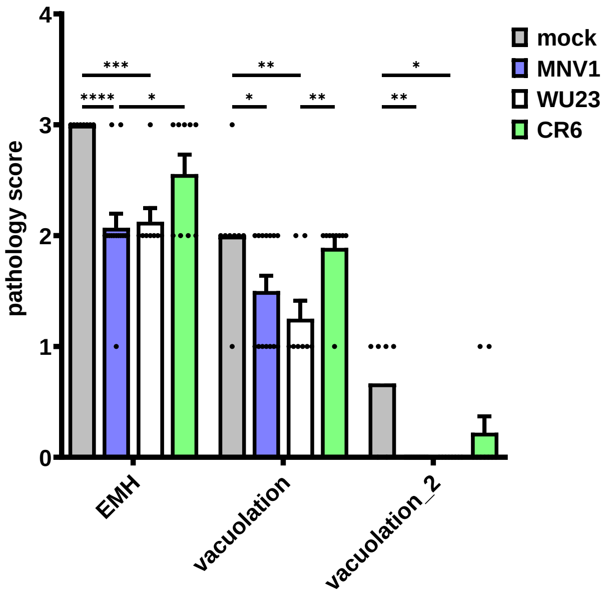

Groups of P3 B6 pups were infected by i.g. with 10^7 $TCID_{50}$ units of MNV1, WU23, CR6, or mock inoculum. At 2 dpi, tissue sections were collected and stained with hematoxylin and eosin. Sections were scored blindly for pathological changes in the indicated segments of the small intestine **(a)**, spleen **(b)**, and liver **(c)**. At least seven mice from at least two independent litters were analyzed for each condition. SI = small intestine; EMH = extramedullary hematopoiesis.

Supplemental Figure 4. Disease severity does not correlate with nonproductive virus uptake into ileal enterocytes.

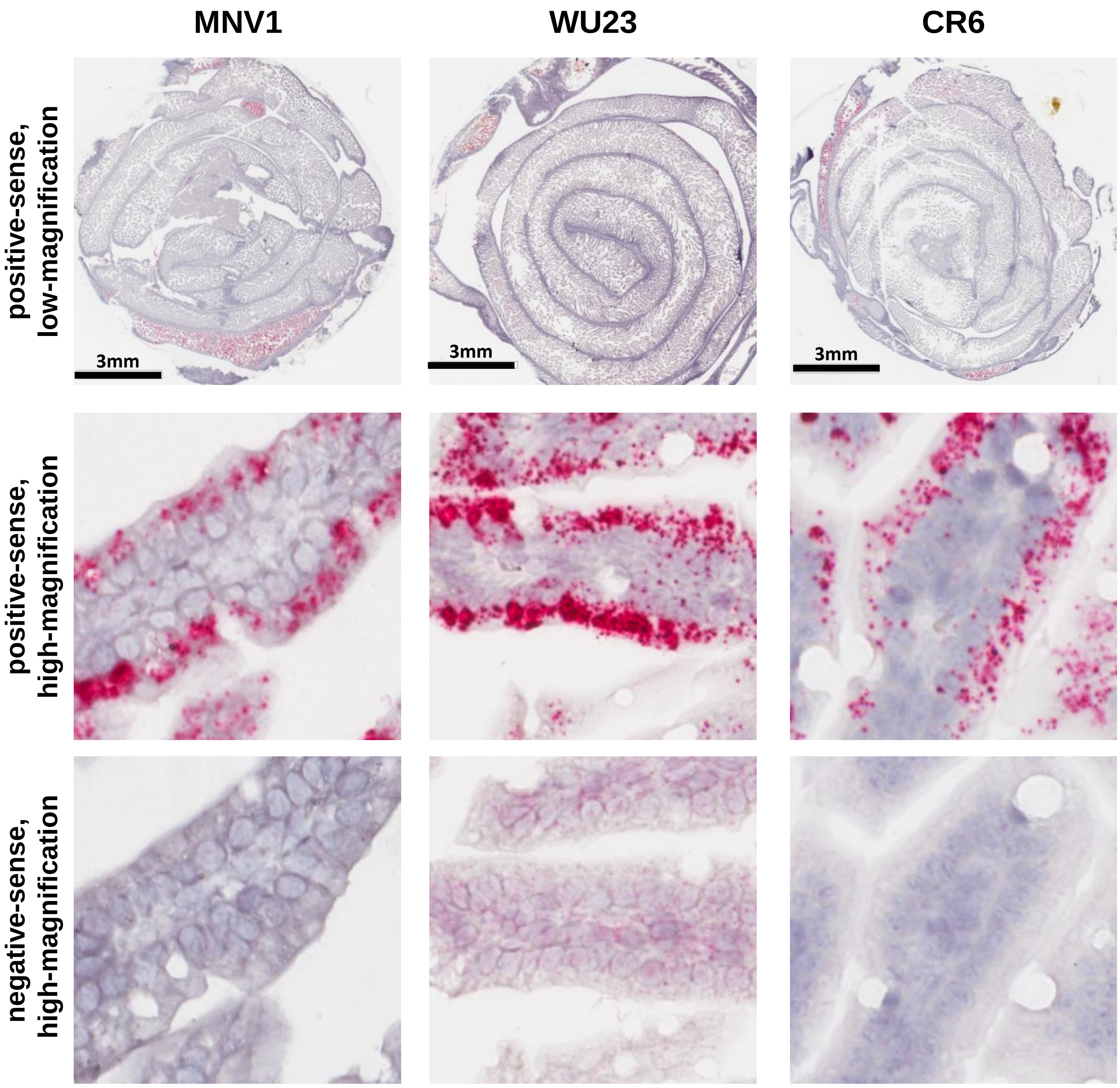

a) Groups of P3 B6 pups were infected by i.g. with 10^8 $TCID_{50}$ units of the indicated virus. At 18 hpi, intestines were harvested, fixed with 10% formalin, embedded and sectioned. Serial sections were hybridized with probes to the positive-sense or negative-sense viral RNA species, as indicated. Tissue sections from at least four mice collected from a minimum of two different litters per condition were analyzed and representative images are shown. Mock-inoculated mice were tested in parallel in every experiment and no viral RNA was detected.

Supplemental Figure 5: Infection dose of 1 TCID50 unit infects B6 pups and is nonlethal in *Ifnar1*$^{-/-}$ pups.

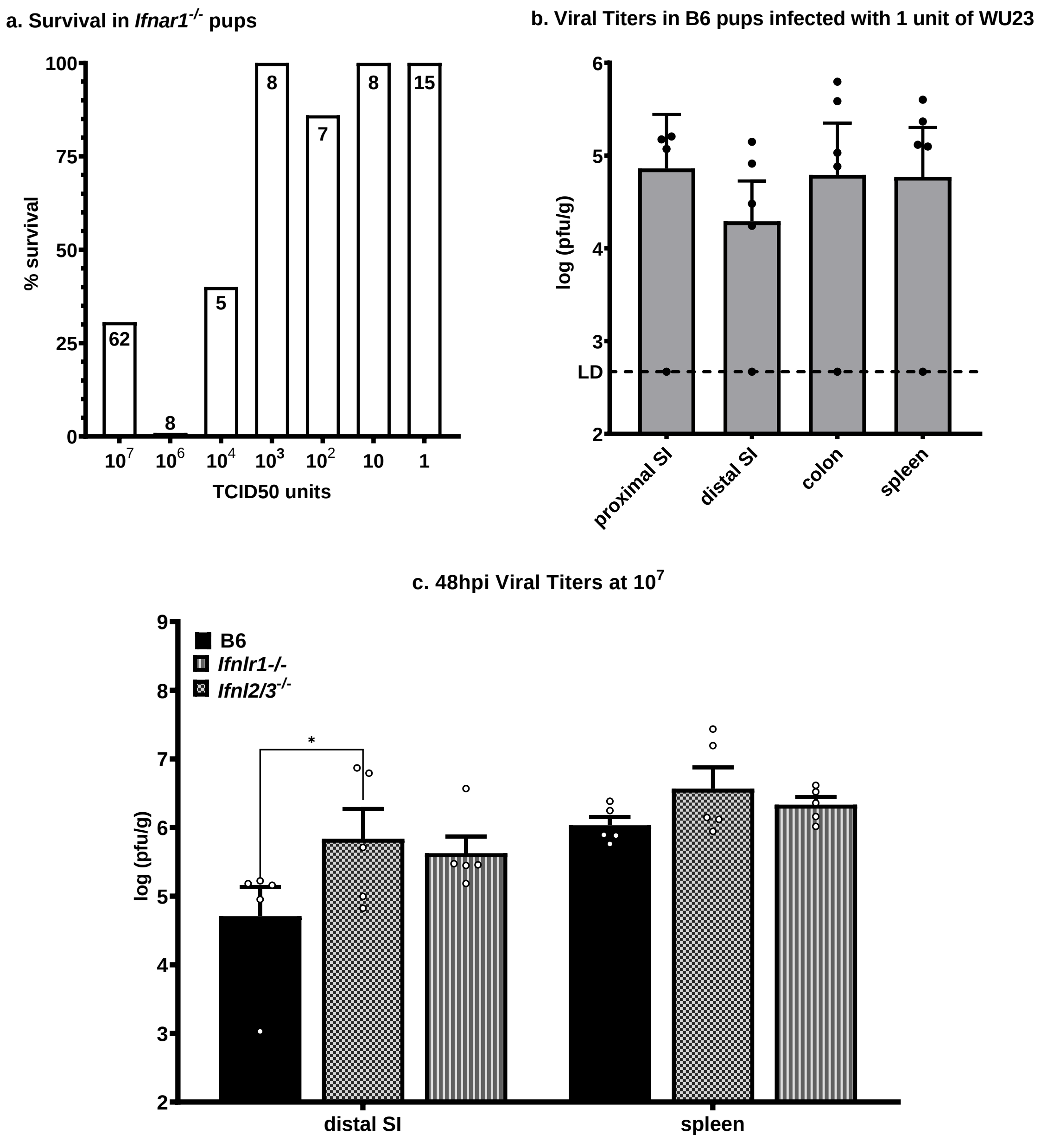

a) Groups of P3 *Ifnar1*$^{-/-}$ pups were infected by i.g. with the indicated dose of WU23. The percentage of pups that survived to 2 dpi is shown. The total number of pups analyzed is indicated within the bar. **b)** Groups of P3 B6 pups were infected i.g. with 1 $TCID_{50}$ unit of WU23. Virus titers at 2 dpi were determined by plaque assay for the proximal small intestine, distal small intestine, colon, and spleen. **c)** Groups of P3 B6, *Ifnlr1*$^{-/-}$ *or Ifnl2/3*$^{-/-}$ pups were infected i.g. with 10^7 $TCID_{50}$ unit of WU23. Virus titers at 2 dpi were determined by plaque assay for the proximal small intestine, distal small intestine, colon, and spleen. At least five pups from at least two independent litters per condition were analyzed. Error bars denote standard errors of mean in all figures.

Supplemental Figure 6: Intestinal pathology in adult *Ifnar1*$^{-/-}$ mice infected with WU23.

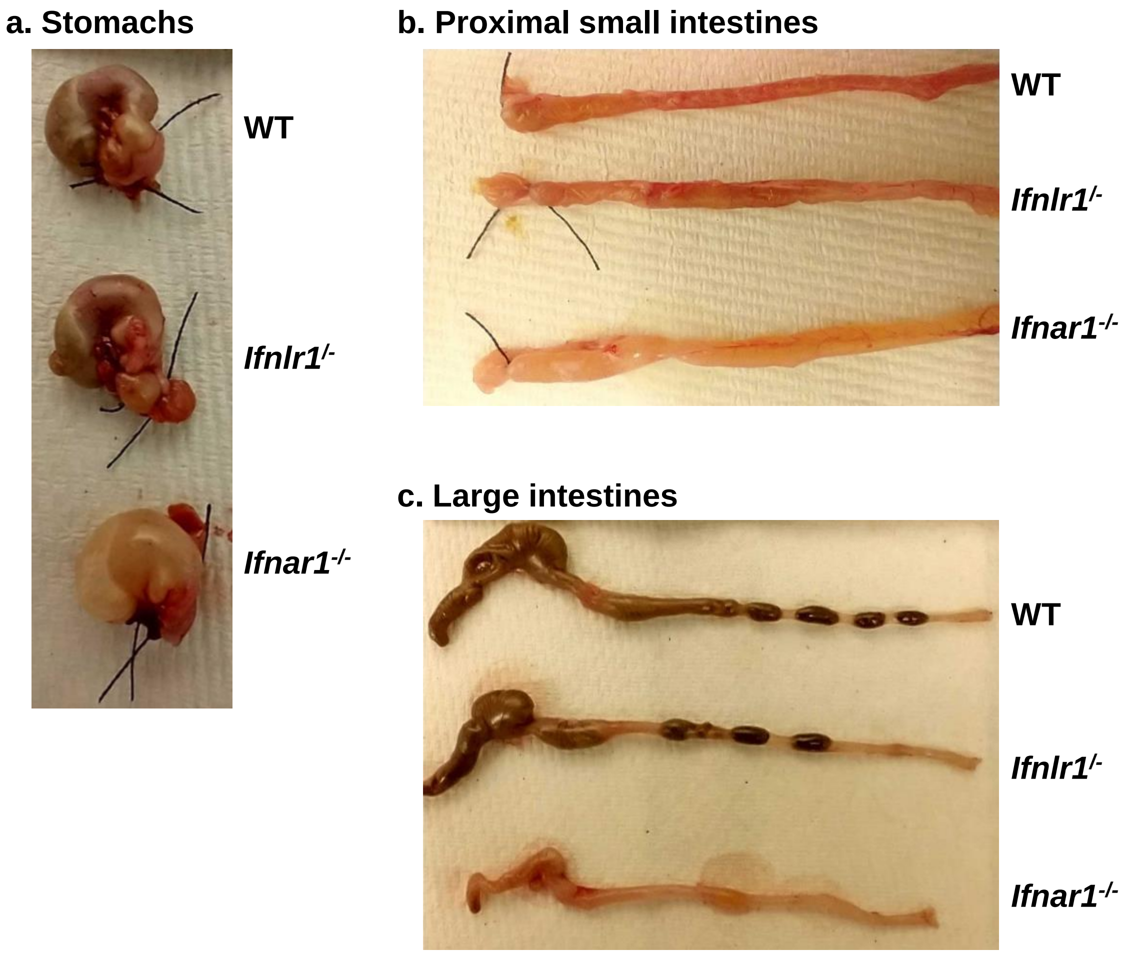

Groups of adult B6, *Ifnar*$^{-/-}$, and *Ifnlr*$^{-/-}$ mice were perorally infected with 10^7 $TCID_{50}$ units of MNV1, WU23, CR6 or mock inoculum and sacrificed at 3 dpi. At least ten mice per condition from three independent experiments were analyzed and representative images of the stomach **(a)**, proximal small intestine **(b)**, and large intestine **(c)** are shown.

Supplemental Figure 7. WU23 stock made from the infectious clone plasmid behaves similarly to uncloned virus stock.

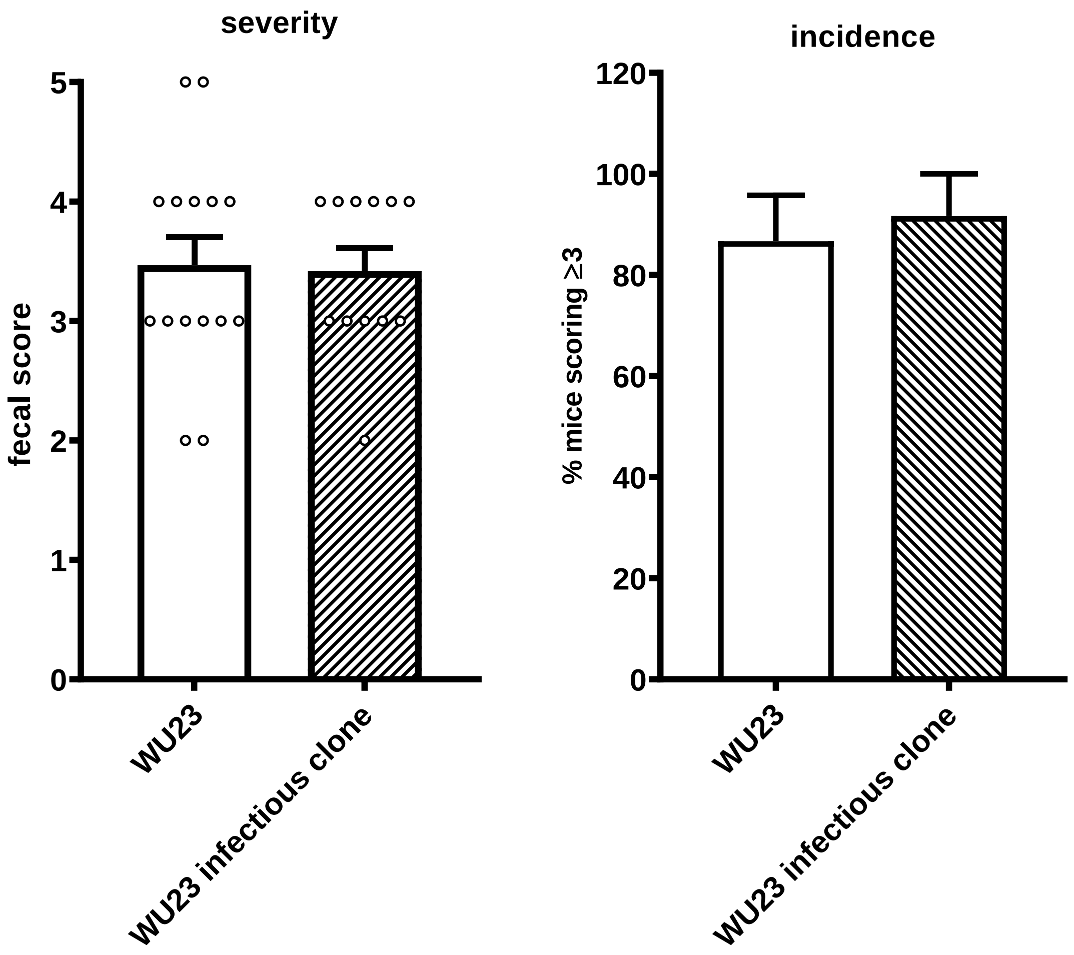

Groups of P3 BALB/c neonatal mice were inoculated with 10^7 $TCID_{50}$ units of passaged WU23 virus stock (WU23) or WU23 virus stock prepared from the infectious clone plasmid (WU23 infectious clone). At least eleven mice from at least two independent litters were analyzed. At 2 dpi, fecal consistency was determined by palpating their abdomens (left graph). The proportion of mice scoring a 3 or above is presented as incidence of diarrhea (right graph). Error bars denote standard errors of mean in all figures.
