# Supplementary material for: Infection of neonatal mice with the murine norovirus strain WU23 is a robust model to study norovirus pathogenesis
Source: Lab Anim (NY). 2023 May 4;52(6):119–29. doi: 10.1038/s41684-023-01166-5 (PMC10234811; doi:10.1038/s41684-023-01166-5)
Supplement: Supplementary file 2 — Reporting Summary [file 41684_2023_1166_MOESM2_ESM.pdf]

nature portfolio

Corresponding author(s): Stephanie Karst

Last updated by author(s): 3/29/2023

Reporting Summary

Nature Portfolio wishes to improve the reproducibility of the work that we publish. This form provides structure for consistency and transparency in reporting. For further information on Nature Portfolio policies, see our Editorial Policies and the Editorial Policy Checklist.

Statistics

For all statistical analyses, confirm that the following items are present in the figure legend, table legend, main text, or Methods section.

n/a	Confirmed	
☐	☒	The exact sample size (*n*) for each experimental group/condition, given as a discrete number and unit of measurement
☒	☐	A statement on whether measurements were taken from distinct samples or whether the same sample was measured repeatedly
☐	☒	The statistical test(s) used AND whether they are one- or two-sided<br>*Only common tests should be described solely by name; describe more complex techniques in the Methods section.*
☒	☐	A description of all covariates tested
☐	☒	A description of any assumptions or corrections, such as tests of normality and adjustment for multiple comparisons
☐	☒	A full description of the statistical parameters including central tendency (e.g. means) or other basic estimates (e.g. regression coefficient) AND variation (e.g. standard deviation) or associated estimates of uncertainty (e.g. confidence intervals)
☐	☒	For null hypothesis testing, the test statistic (e.g. *F*, *t*, *r*) with confidence intervals, effect sizes, degrees of freedom and *P* value noted<br>*Give P values as exact values whenever suitable.*
☒	☐	For Bayesian analysis, information on the choice of priors and Markov chain Monte Carlo settings
☒	☐	For hierarchical and complex designs, identification of the appropriate level for tests and full reporting of outcomes
☒	☐	Estimates of effect sizes (e.g. Cohen's *d*, Pearson's *r*), indicating how they were calculated

Our web collection on statistics for biologists contains articles on many of the points above.

Software and code

Policy information about availability of computer code

Data collection	No software was used to collect data.
Data analysis	GraphPad Prism 9 was used for statistical analysis.

For manuscripts utilizing custom algorithms or software that are central to the research but not yet described in published literature, software must be made available to editors and reviewers. We strongly encourage code deposition in a community repository (e.g. GitHub). See the Nature Portfolio guidelines for submitting code & software for further information.

Data

Policy information about availability of data

All manuscripts must include a data availability statement. This statement should provide the following information, where applicable:

- Accession codes, unique identifiers, or web links for publicly available datasets
- A description of any restrictions on data availability
- For clinical datasets or third party data, please ensure that the statement adheres to our policy

The datasets generated and analyzed in the current study are available from the corresponding authors on reasonable request.

Field-specific reporting

Please select the one below that is the best fit for your research. If you are not sure, read the appropriate sections before making your selection.

☒ Life sciences ☐ Behavioural & social sciences ☐ Ecological, evolutionary & environmental sciences

For a reference copy of the document with all sections, see nature.com/documents/nr-reporting-summary-flat.pdf

Life sciences study design

All studies must disclose on these points even when the disclosure is negative.

Sample size	For quantitative analyses, we used power analysis to obtain a power of 80% at alpha = 0.05.
Data exclusions	For fecal scoring studies, mice that did not defecate upon palpation of their abdomens were excluded from analysis because it was not possible to score fecal consistency.
Replication	We clearly state the number of experimental replicates in each figure legend or in supplemental material, as indicated. For quantitative analyses, we average data for all replicates. For visual analyses, we selected the most representative images available. In all cases, findings were reliably reproduced.
Randomization	Mice were age- and sex-matched for all animal experiments.
Blinding	Mouse experiments were not performed in a blinded fashion.

Reporting for specific materials, systems and methods

We require information from authors about some types of materials, experimental systems and methods used in many studies. Here, indicate whether each material, system or method listed is relevant to your study. If you are not sure if a list item applies to your research, read the appropriate section before selecting a response.

Materials & experimental systems

n/a	Involved in the study	
☒	☐	Antibodies
☐	☒	Eukaryotic cell lines
☒	☐	Palaeontology and archaeology
☐	☒	Animals and other organisms
☒	☐	Human research participants
☒	☐	Clinical data
☒	☐	Dual use research of concern

Methods

n/a	Involved in the study	
☒	☐	ChIP-seq
☒	☐	Flow cytometry
☒	☐	MRI-based neuroimaging

Eukaryotic cell lines

Policy information about cell lines

Cell line source(s)	BV2 (ATCC), HEK293T (provided by Fanxiu Zhu, Florida State University)
Authentication	Cell lines were not authenticated.
Mycoplasma contamination	All cell lines tested negative for mycoplasma contamination.
Commonly misidentified lines (See ICLAC register)	These cell lines are not commonly misidentified.

Animals and other organisms

Policy information about studies involving animals; ARRIVE guidelines recommended for reporting animal research

Laboratory animals	C57BL/6J(Jackson no. 000664), BALB/c (Charles River no. 028), C57BL/6J-Ifnar1-/- (Jackson no. 010830), C57BL/6J-Ifnlr1-/- (Baldridge lab, Washington University in St. Louis), C57BL/6J-Ifnl2/3-/- (Baldridge lab, Washington University in St. Louis)
Wild animals	The study did not involve wild animals.
Field-collected samples	The study did not involve samples collected from the field.

Ethics oversight	All animal experiments were performed in strict accordance with federal and university guidelines and approved by the Institutional Animal Care and Use Committee at the University of Florida (study numbers 20190632, 201810473, 202110473, and 202200000065).

Note that full information on the approval of the study protocol must also be provided in the manuscript.
